# *PaPro1* and *IDC4*, Two Genes Controlling Stationary Phase, Sexual Development and Cell Degeneration in *Podospora anserina*

**DOI:** 10.3390/jof4030085

**Published:** 2018-07-11

**Authors:** Valérie Gautier, Laetitia Chan Ho Tong, Tinh-Suong Nguyen, Robert Debuchy, Philippe Silar

**Affiliations:** 1 Sorbonne Paris Cité, Laboratoire Interdisciplinaire des Energies de Demain (LIED), University Paris Diderot, 75205 Paris, France; valerie.gautier@univ-paris-diderot.fr (V.G.); chan.laetitia@gmail.com (L.C.H.T.); tsnguyen91@yahoo.fr (T.-S.N.);; 2 Institute for Integrative Biology of the Cell (I2BC), CEA, CNRS, University Paris-Sud, Université Paris-Saclay, CEDEX, 91198 Gif-sur-Yvette, France; Robert.DEBUCHY@i2bc.paris-saclay.fr

**Keywords:** multicellular fruiting bodies, fungal development, developmental mutants, *Podospora anserina*, perithecium

## Abstract

Filamentous fungi frequently undergo bistable phenotypic switches. Crippled Growth of *Podospora anserina* is one such bistable switch, which seems to rely upon the mis-activation of a self-regulated PaMpk1 MAP kinase regulatory pathway. Here, we identify two new partners of this pathway: PaPro1, a transcription factor orthologous to *Sordaria macrospora* pro1 and *Neurospora crassa* ADV-1, and IDC4, a protein with an AIM24 domain. Both *PaPro1* and *IDC4* regulate stationary phase features, as described for the other actors of the PaMpk1 signaling pathway. However, PaPro1 is also involved in the control of fertilization by activating the transcription of the HMG8 and the mating type transcription factors, as well as the sexual pheromones and receptor genes. The roles of two components of the STRIPAK complex were also investigated by inactivating their encoding genes: *PaPro22* and *PaPro45*. The mutants of these genes were found to have the same phenotypes as *PaPro1* and *IDC4* mutants as well as additional phenotypes including slow growth, abnormally shaped hyphae, pigment accumulation and blockage of the zygotic tissue development, indicating that the STRIPAK complex regulates, in addition to the PaMpk1 one, other pathways in *P. anserina*. Overall, the mutants of these four genes confirm the model by which Crippled Growth is due to the abnormal activation of the PaMpk1 MAP kinase cascade.

## 1. Introduction

Phenotypic switches visible as “sectors” in the thallus are of frequent occurrence in filamentous fungi [1]. Their etiology is often unknown, and sectors may originate from genetic or epigenetic modifications. The filamentous fungus *Podospora anserina* undergoes such bistable phenotypic switch when grown on medium containing yeast extract (YE) [2,3]. Indeed, when cultivated on minimal medium lacking YE (M2 medium), it grows profusely until it dies from senescence. On the contrary, when cultivated on M2 medium with YE (M2 + YE), it frequently presents sectors of the so called “crippled growth” (CG), characterized by slower growth rate, accumulation of pigment, lack of aerial hyphae and fruiting bodies. Early analyses [2,3] showed that CG could be triggered when the mycelium is incubated into stationary phase and replicated onto M2 + YE. The reverse switch from CG to a normal growth (NG) can be obtained by stressing the cultures or by growing them on M2 or germination medium [2,3]. Cell fusion (anastomosis) experiments showed that CG could be transmitted to NG strain by a cytoplasmic and infectious factor, called *C*, in the absence of mitochondrial DNA modification [3]. This strongly suggested that this bistable phenotypic switch is an epigenetic prion-like phenomenon rather than due to a genetic mutation.

To understand the phenomenon, a genetic approach was chosen and mutants affecting CG positively (*PDC* mutants for Promoting the Development of CG) and negatively (*IDC* mutants for Impairing the Development of CG) were selected [2]. Two groups of *IDC* mutants were defined [2]. Some were crucial to CG and all had the same phenotypes: they lacked melanin, aerial hyphae and were female sterile. They were called the “pink” *IDC* mutants, because they accumulated upon long incubation a pink pigment instead of the dark-green melanin produced normally by the fungus. The “non-pink” mutants displayed a wider range of phenotypes, could still transmit CG in anastomosis experiments and were hypothesized to act through stress. On the contrary, the “pink” IDC mutants were postulated to be affected in key elements enabling the production of the cytoplasmic and infectious factor *C*.

Many of the “pink” *IDC* mutants have already been characterized; especially, the genes affected in these *IDC* mutants have been identified. The first characterized ones encode the PaASK1 MAP kinase kinase kinase (MAPKKK) [4] and the PaNox1 catalytic subunit of the NADPH oxidase 1 (Nox1) [5], leading to the proposal that CG originates from the abnormal activation of a positively self-regulated MAP kinase (MAPK) cascade. In the model, PaNox1 would be implicated in a reactive oxygen species (ROS)-mediated activation of PaMpk1 MAPK cascade in response to starvation [2,5]. This model was further substantiated by the identification of the PaMKK1 MAP kinase kinase (MAPKK) and the PaMpk1 MAPK as crucial for CG development [6]. Based on the phenotypes of the “pink” *IDC* mutants, it was deduced that the cascade is involved in the control of the entry into stationary phase [4]. Indeed, melanin, aerial hyphae and development of fruiting bodies are features typical of stationary phase. In addition, the mutants were later shown to be unable to undergo anastomoses, another feature occurring in early stationary phase [7]. On medium lacking YE, the cascade would thus be active only during stationary phase. On M2 + YE, the cascade would also activate randomly in growing cells, triggering CG.

Additional partners of the signaling pathway were identified thanks to the analysis of the other “pink” *IDC* mutants: the regulatory (PaNoxR) and docking (PaNoxD) subunits of the Nox1 complex [8,9], IDC2 and IDC3, two proteins with conserved cysteines crucial for their function and that are supposed to be part of the pathway that connects the ROS-generating Nox1 complex with the MAPK [10], the PaPsr1/PaWhi2 proteins whose role in the pathway is presently unknown [11]. A role of a second MAPK pathway, composed of PaTLK2, PaMKK2 and PaMpk2, was also evidenced [12]. Indeed, it was shown that the PaMpk2 pathway is required for activity of the PaMpk1 one [12]. Two additional factors have been identified as affected in some *IDC* mutants: PaSo and IDC1 [7,13]. In *Neurospora crassa* and *Sordaria macrospora*, So (also known as pro40 in *S. macrospora*), the orthologue of PaSo, and HAM-5, the orthologue of IDC1, were shown to be the scaffold for the PaMpk1 and PaMpk2 MAP kinase pathways, respectively [14,15,16,17,18]. Presently, only three “pink” *IDC* mutants isolated by Haedens et al. [2] remain to be characterized: *IDC*^508^, *IDC*^510^ and *IDC*^511^. Here, we identify by complete genome sequencing the genes affected in these three mutants and begin the analysis of their role in the MAPK cascades and CG.

## 2. Materials and Methods

### 2.1. Strains and Culture Conditions

All the mutant strains used in this study derived from the “S” (uppercase S) wild-type strain that was used for sequencing [19,20]. Standard culture conditions, media and genetic methods for *P. anserina* have been described [21,22] and are available at http://podospora.i2bc.paris-saclay.fr.

### 2.2. Mutation Identifications

To identify the gene mutated in *IDC*^508^, *IDC*^510^ and *IDC*^511^, we backcrossed the mutants for five additional generations with the parental wild-type strain in order to eliminate mutations without involvement in the phenotype that may have been created after UV treatment (Silar 2013). DNA was extracted from one randomly selected progeny of the final backcrossed presenting the *IDC* phenotype. The genomic DNA was subjected to complete sequencing with the Illumina technology. For the Illumina sequencing, this work has benefited from the facilities and expertise of the high throughput sequencing platform of IMAGIF (Centre de Recherche de Gif—www.imagif.cnrs.fr). Custom-made libraries had 300 bp inserts and sequencing was 76-bp paired-end. Coverage was ~90 fold. Sequence reads were mapped onto the last version of the reference genome of the S strain [20] and potential mutations were detected using the Galaxy web server (https://usegalaxy.org/).

### 2.3. Gene Deletions

To construct the deleted alleles of *PaPro1*, *IDC4*, *PaPro22* and *PaPro45*, a PCR fusion strategy was adopted [22] using the primers of Table S1.

To construct the deletion cassette for *IDC4* (Pa_2_230), a geneticin resistance marker was fused by PCR with sequences 5′ and 3′ of the *IDC4* gene. Two ~500-bp regions located upstream and downstream of the Pa_2_230 CDS were amplified from genomic DNA using primers (Table S1) 5′ Pa_2_230A and 5′ Pa_2_230B for the upstream region and 3′ Pa_2_230C and 3′ Pa_2_230D for the downstream one. The primers used for this amplification contained additional bases allowing fusion with the geneticin resistance marker. The geneticin marker was amplified from the pBC-geneticin vector [7] with primer mk_2_230E and mk_2_230F. In a second step, two amplifications were performed using a mix of either one of the upstream or downstream sequences with the geneticin-amplified DNA and the distal primers 5′ Pa_2_230A/mk_2_230F and mk_2_230E/3′ Pa_2_230D to obtain two fusion PCR products: 5′ region/gen and gen/3′ region, respectively. The mixture of these two PCR products was used to transform a ∆*mus-51::phleomycinR* strain. 10 transformants resistant to geneticin were obtained thanks to three crossing-over events between the transformed PCR products and the genome. Five transformants were crossed to the wild-type strain and homokaryotic *mat+ IDC4*^∆^ and *mat− IDC4*^∆^ ascospores devoid of the ∆*mus-51::phleomycinR* marker were identified. Two F2 progeny were verified that they were indeed properly deleted for the *IDC4* gene by Southern Blotting (Figure S1). Both had the expected pattern.

*PaPro1* was replaced with a geneticin resistance marker using primers 5′Pa_1_10140A and 5′Pa_1_10140B and 3′Pa_1_10140C and 3′Pa_1_10140D (Table S1). The geneticin marker was amplified from the pBC-geneticin vector with primer mk_1_10140E and mk_1_10140F (Table S1). The 5′ region/gen and gen/3′ regions were co-transformed into the ∆*mus-51::phleomycinR* strain and 11 transformants were recovered. Five were crossed with wild type and one *mat+ PaPro1*^∆^ and one *mat− PaPro1*^∆^ ascospores devoid of the ∆*mus-51::phleomycinR* marker were analyzed by Southern blotting (Figure S1). Both had the expected pattern.

*PaPro22* was replaced with a hygromycin B resistance marker using primers 5′Pa_2_9440A and 5′Pa_2_9440B and 3′Pa_2_9440C and 3′Pa_2_9440D (Table S1). The hygromycin B marker was amplified from the pBC-hygro vector with primer mk_2_9440E and mk_2_9440F (Table S1). The 5′ region/gen and gen/3′ regions were co-transformed into the ∆*mus-51::phleomycinR* strain and 11 transformants were recovered. All had the same slow-growing phenotype. Two were crossed with wild type and *mat+ PaPro22*^∆^ and *mat− PaPro22*^∆^ ascospores devoid of the ∆*mus-51::phleomycinR* marker were selected for further analysis.

*PaPro45* was replaced with a hygromycin B resistance marker using primers 5′Pa_1_15490A and 5′Pa_1_15490B and 3′Pa_1_15490C and 3′Pa_1_15490D (Table S1). The hygromycin B marker was amplified from the pBC-hygro vector with primer mk_1_15490E and mk_1_15490F (Table S1). The 5′ region/gen and gen/3′ regions were co-transformed into the ∆*mus-51::phleomycinR* strain and 13 transformants were recovered. All had the same slow-growing phenotype. Two were crossed with wild type and *mat+ PaPro1*^∆^ and *mat*− *PaPro1*^∆^ ascospores devoid of the ∆*mus-51::phleomycinR* marker were selected for further analysis.

### 2.4. Gene Complementation

Complementation of *PaPro1*^∆^ was achieved by co-transforming plasmid GA0AA351CH02 with the pBC-hygro vector [23] into the *PaPro1*^∆^. Plasmid GA0AA351CH02 was obtained during the *P. anserina* genome sequencing project (Espagne et al., 2008) and carried a 3-kb insert encompassing the wild-type *PaPro1^+^* allele and no neighboring gene. Two hygromycin B-resistant transformants having a near wild-type phenotype were recovered and were crossed with wild-type. Progeny analysis showed that restoration of a near wild-type phenotype co-segregated with the resistance to hygromycin B. One *PaPro1*^∆^ progeny carrying a complementing transgene was crossed with *IDC*^511^. In this cross, we recovered progeny carrying *IDC*^511^ and the transgene. These progeny also has a near wild-type phenotype.

Complementation of the *IDC4* mutants was achieved by co-transforming a PCR product obtained by using primers IDC^508^-left and IDC^508^-right (Table S1) with the pBC-hygromycin vector into *IDC4*^Δ^ and *IDC*^508^. In several independent transformations, we obtained very few transformants for both mutants, suggesting some transformation impairment in *IDC4*^Δ^ and *IDC*^508^. However, among the three transformants recovered with *IDC4*^Δ^, one had a wild-type phenotype. When crossed with *IDC*^508^, it produced a progeny in which the wild-type phenotype co-segregated with resistance to hygromycin B. Analysis of this progeny showed that the transgene enabling the restoration of a wild-type phenotype in *IDC4*^Δ^ also permitted to restore a wild-type phenotype in *IDC*^508^.

### 2.5. Plasmid Constructions for mCherry-Tagging of IDC4

The 3′-end of the IDC4 gene was amplified with primers “XhoI 508 tag for” and “Hind3 508 tag rev” (Table S1). This resulted in a 500–bp fragment in which the stop codon of the IDC4 CDS was replaced with a glycine codon. This fragment was cloned in frame with the mCherry CDS in the pmCherry_hyg [8] to yield pIDC4-mCherry. This plasmid was used to transform the ∆*mus-51::phleomycinR* strain. A hygromycin B-transformant in which the plasmid had integrated by a single crossing over was recovered. In this transformant, the mCherry CDS was put in frame with the IDC4 CDS. This transformant had a wild-type phenotype and exhibited a red fluorescence when excited at 505 and 555 nm, showing that the IDC4-mCherry fusion protein was functional. This transformant was crossed with a strain carrying the mito-GFP transgene [24]. In the progeny, strains carrying both transgenes expressing IDC4-mCherry and mito-GFP were recovered.

### 2.6. RT-qPCR Experiments

Vegetative cultures for RNA preparation were made on Petri dishes containing M2 minimal medium and covered with a cellophane sheet (cat#1650963, Bio Rad Hercules, USA). These cultures were inoculated with nine implants from WT *mat+*, *PaPro1*^∆^ *mat+,* WT *mat−* or *PaPro1*^∆^ *mat−* strains. Dishes were placed at 27 °C under constant light and were removed from the incubation room at 96 h, at which time *P. anserina* was competent for fertilization [25]. Mycelia were harvested and RNAs were extracted as described previously [26]. RNA quality was checked by gel electrophoresis. When possible, primers were designed on two consecutive exons (Table S1). A non-reverse transcribed (NRT) control was systematically performed on a pool of replicates to ensure that the Cq was above the Cq obtained from corresponding reverse transcribed RNAs. For RNAs without introns, NRT control was performed for each replicate sample to ensure that the Cq was above the Cq obtained from corresponding reverse transcribed RNAs. Experiments were run with six biological replicates of each strain, and biological replicate was run in technical triplicates (Table S2). Normalization genes were selected from a pool of 9 housekeeping genes using geNorm [27] (Table S3). RT-qPCR normalization according to the ΔΔCt method, standard error and 95% confidence interval calculations, and statistical analyses were performed using REST 2009 software (Qiagen, Hilden, Germany) [28] (Table S4). RT-qPCR experiments were MIQE compliant [29].

### 2.7. Microscopy

Observations were performed on 3-days old cultures grown on M2 medium with an inverted Leica DMI6000 microscope, equipped with a 100× oil objective. GFP was excited between 440 and 490 nm and emission collected between 500 and 550 nm; mCherry was excited between 505 and 555 nm and emission collected between 570 and 620 nm. Images were analyzed with ImageJ (Rasband, W.S., ImageJ, U.S. National Institutes of Health, Bethesda, Maryland, USA, http://imagej.nih.gov/ij/, 1997–2014).

### 2.8. Phylogenetic Analysis

Orthologous and paralogous genes present in fungal genomes were searched by BLAST using the default parameters with the IDC4 protein as queries. Alignment was made with MAFFT [30] and manually refined. This alignment was used to construct a phylogenetic tree using the maximum likelihood method (PhyML software [31]) and transferred to the iTOL server for visualization [32]. Bootstrap values are expressed as percentages of 100 replicates.

### 2.9. FIMO Analysis

The intergenic regions upstream of selected genes (i.e., the sequence located upstream the genes translation start codon and the start/stop codon of the upstream genes) was searched using the FIMO web server (http://meme-suite.org/doc/fimo.html) with 10^−4^ and 10^−3^ *p*-values.

### 2.10. Protein Extraction and Western Blot Analysis

Phosphorylation of PaMpk1 was analyzed as described in [6]. Western blots were made and analyzed for three independent protein extractions.

## 3. Results

### 3.1. Identification of the Genes Affected in IDC^508^, IDC^510^ and IDC^511^

To identify the genes affected in the three remaining *IDC* mutants, for whom no molecular data are yet available, we completely sequenced their genomes as described in the Materials and methods section. In the *IDC*^508^ genome, we found three mutations near the centromere of chromosome 2 onto which the mutated gene was mapped [2]. One was in an intergenic region, one was a conservative change of a valine codon (GTA to GTG) in *Pa_2_1570* that encodes a glycoside hydrolase of family 16 and the last one affected two contiguous nucleotides of *Pa_2_230*, resulting in a premature stop codon at position 73. This latter mutation was validated as causing the IDC phenotype (see below). *Pa_2_230* was thus renamed *IDC4*.

In the *IDC*^510^ genome, we identified a mutation in the *PaTLK2* MAPKKK gene changing codon n°362 encoding glutamine into a stop codon. The phenotype of *IDC*^510^ is identical to the one of the deletion of *PaTLK2*, including lack of ascospore germination [2,12], validating the mutation in this gene as the one responsible for the IDC phenotype. This mutant was thus not further investigated because the *PaTLK2* gene has already been extensively studied [12].

In the *IDC*^511^ genome, we identified three mutations. Two were located in intergenic regions of chromosome 1, while the third one changed the arginine codon n°156 into a stop codon in *Pa_1_10140*, a coding sequence (CDS) also located on chromosome 1. This CDS is an orthologue of the *Sordaria macrospora pro1* gene [33] and was thus renamed *PaPro1*. This latter mutation was validated as causing the IDC phenotype (see below).

### 3.2. Inactivation of PaPro1 in IDC^511^ Creates the IDC Phenotype

**Figure 1:**
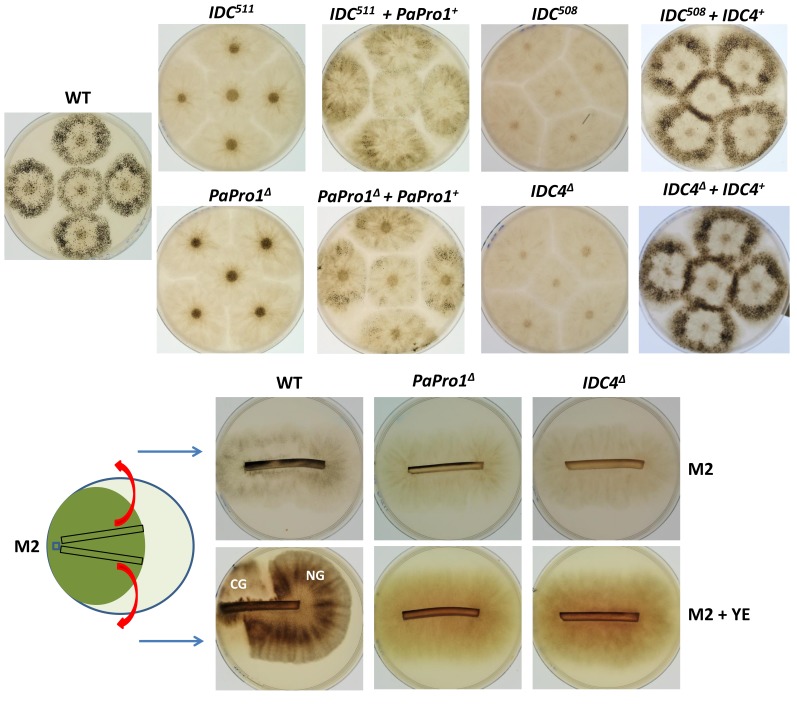
IDC phenotype of *PaPro1* and *IDC4* mutants. *mat*+/*mat*− heterokaryotic mycelia were inoculated onto M2 medium in Ø = 8 cm Petri plates and incubated for one week, at which time the pictures were taken. The fruiting bodies in the wild type and complemented strains are visible as small black dots. The CG tests at the bottom were made by re-inoculating (red arrows) mycelium slices grown on M2 for seven days and collected as indicated on the left scheme onto fresh media with or without yeast extract. Photos were taken five days after reinoculation. Only the wild type shows CG that originated from hyphae that were in stationary phase. NG, normal Growth.

To prove that inactivation of *PaPro1* was indeed responsible for the IDC phenotype, we deleted the *PaPro1* gene using the split marker method (see Section 2) and the deleted strain was validated by Southern blot analysis (Figure S1). The deletion of *PaPro1* (*PaPro1*^Δ^) created a typical “pink” IDC phenotype (Figure 1). Reintroduction of the wild-type allele in the *PaPro1*^Δ^ and *IDC*^511^ strains restored pigment production and fertility (Figure 1), finally demonstrating that *PaPro1* is the gene affected in *IDC*^511^ (Figure 1). However, complementation was partial because the fruiting bodies in the complemented strains were smaller and in lower amounts than in the wild type (Figure 1). This is frequently observed since DNA integration for transformation occurs randomly in the *P. anserina* genome and correct expression of the transgene may not occur.

**Figure 2:**
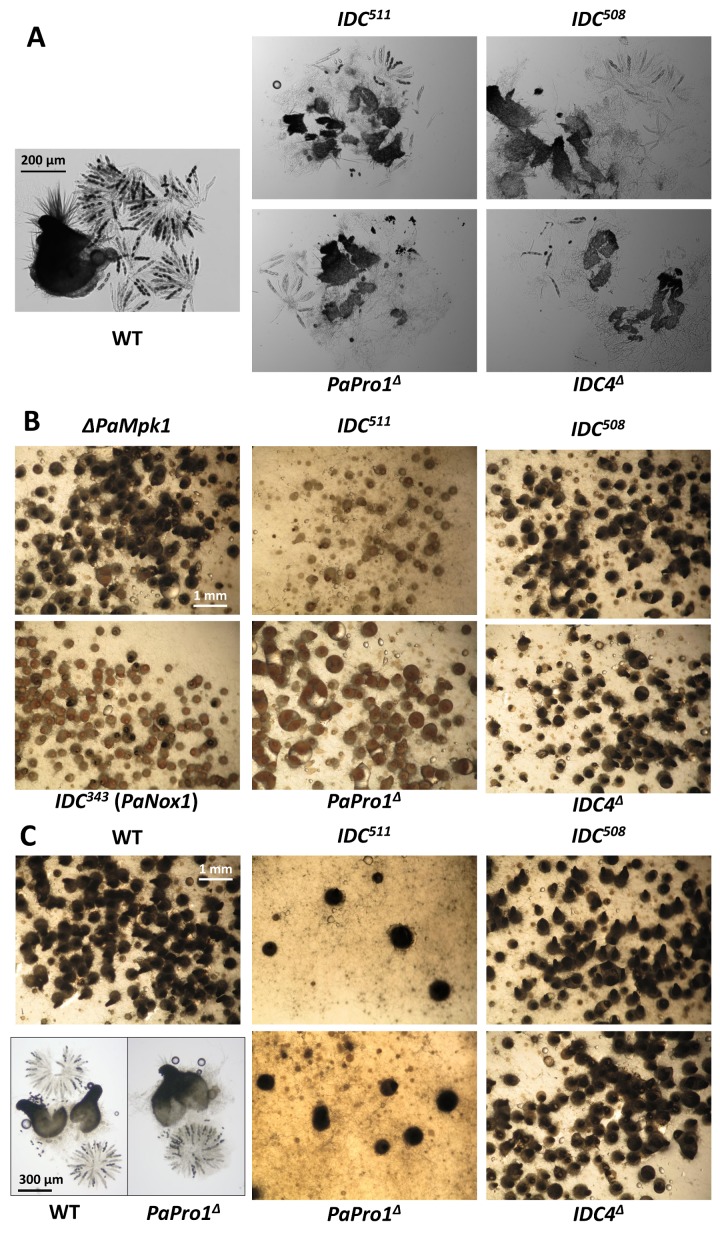
Fertility defect of the *IDC4* and *PaPro1* mutants. (**A**) Grafting experiments show that *IDC4* and *PaPro1* are required in the mycelium during fruiting body maturation. Two-day-old wild type perithecia (WT) grafted onto wild-type mycelia continued their development and produced many asci. On the contrary, those grafted onto *IDC4* or *PaPro1* mutant strains failed to mature properly, i.e., remained small and differentiated few abortive asci. (**B**) Mosaics of the indicated strains with *psk1-193* of opposite mating show that *IDC4* is dispensable in the fruiting body, since the mosaic differentiated pigmented fruiting bodies having the *IDC4* mutants as the maternal parent. On the contrary, *PaPro1* is necessary in the fruiting body since only unpigmented perithecia were produced. The controls are the Δ*PaMpk1* mutant used to show that *PaMpk1* is dispensable in the developing fruiting body and the *IDC*^343^ mutant of *PaNox1* used to show that *PaNox1* is required in the developing fruiting body [5]. Note that in the case of the *PaNox1* mutants a few perithecia with a pigmented neck were obtained as previously described [5], while none were produced in the case of the two *PaPro1* mutants. (**C**) mosaics with the Δ*mat* mutant resulted in abundant ascospore-bearing fruiting body production in the case of the *IDC4* mutants, confirming that *IDC4* is dispensable in the fruiting body, but also for fertilization. On the contrary, few abnormal and/or few ascospore-bearing normal-looking perithecia were obtained with both *PaPro1* mutants, showing that *PaPro1* is required for fertilization and/or at an early stage during formation in the zygotic tissue of the fruiting body. Pictures at the bottom left show that rosettes of asci are like those of the wild type in the few perithecia that developed in the trikaryons made with *PaPro1* mutants.

Fine analysis of the *PaPro1*^Δ^ and *IDC*^511^ phenotypes showed that both *PaPro1* mutants had exactly the same defects, indicating that the *PaPro1* allele present in *IDC^511^* was null. This was expected since the arginine codon n°157 affected in *IDC*^511^ lies between the GAL4-like Zn(II)2Cys6 binuclear cluster DNA-binding domain and the transactivation domain [34]. A stop codon at position 157 would thus result in a protein lacking the transactivation domain. Both *PaPro1* mutants lacked CG, melanin pigments and aerial hyphae (Figure 1), were female sterile (Figure 1) and unable to engage anastomosis (Figure S2). Microscopy examination of one week mycelia showed that spermatia and ascogonia were differentiated by the *PaPro1* mutants. Fertility defect of the *PaPro1* mutants was more thoroughly analyzed by grafting and mosaic analyses [35]. Two-day-old developing wild-type perithecia grafted onto *PaPro1*^Δ^ and *IDC*^511^ mycelia did not continue their development as only one out of 58 grafted fruiting bodies matured partially (Figure 2A). On the contrary, 30 out of 32 perithecia continued their development when grafted onto wild-type mycelium. This showed that *PaPro1* was required in the mycelium for proper fruiting body maturation. Like for the *IDC*^343^ mutant of PaNox1, mosaics of *PaPro1*^Δ^ and *pks1-193* of opposite mating type, as well as that of *IDC*^511^ and *pks1-193*, produced only unpigmented fruiting bodies (Figure 2B), showing that all developing fruiting bodies had *pks1-193* as their maternal parent (as a control, mosaics with Δ*PaMpk1 and pks1-193*, which is dispensable in the fruiting body, differentiated both unpigmented and pigmented fruiting bodies). This showed that *PaPro1* was required in the fruiting body. This was further analyzed by making mosaics with the Δ*mat* strain (Figure 2C). Being devoid of its mating type, this strain is unable to engage fertilization, but can provide maternal tissues for the development of the fruiting body [35]. Very few mature ascospore-bearing perithecia were recovered from *PaPro1*^Δ^ *mat+* × *PaPro1*^Δ^ *mat−* × Δ*mat* and *IDC*^511^ *mat+* × *IDC*^511^ *mat−* × Δ*mat* trikaryons; usually 1–5 perithecia were obtained by Petri plate. This suggested that *PaPro1* was required very early for the development of the zygotic tissues, possibly at the stage of fertilization. However, development proceeded normally in the few perithecia that succeeded to perform fertilization, suggesting that latter stages of the zygotic tissue maturation did not require *PaPro1*. Fertility of a subset of the *IDC* mutants can be rescued by adding paper as a slowly digesting carbon source [5,7,10,11]. As seen on Figure S3, both *PaPro1* mutants could not be rescued by addition of paper, as observed for the *PaMpk1* mutant and unlike the *PaNox1* one. Note that the *PaPro1* mutants were fully able to differentiate the appressorium-like structures that are involved in cellophane penetration [9], but, like some other IDC mutants, were impaired in hyphal interference (Figure S4), a defense mechanism exhibited by *P. anserina* when encountering another filamentous fungus [36,37].

### 3.3. Transcription of PaPro1 Is Not Regulated by the MAPK Pathways

**Figure 3:**
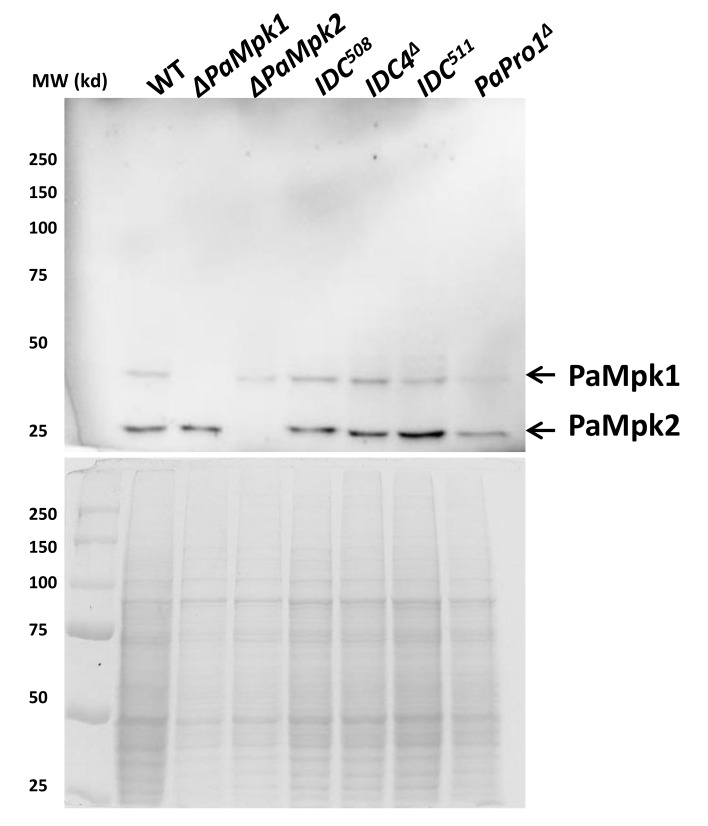
Phosphorylation of PaMpk1 and PaMpk2 is not altered in the *PaPro1* and *IDC4* mutants. Proteins were extracted from 48-h-old mycelia, separated on a 12% acrylamide gel and probed with an antiphospho-MAPK antibody (Top). Comassie blue-stained gel of the same extracts as loading control is at the bottom. Extracts from Δ*PaMpk1* and Δ*PaMpk2* mutants were loaded as controls. No difference in the amount of phosphorylation is detected for PaMpk1 and PaMpk2 phosphorylation.

Analysis of microarray data [38] showed that *PaPro1* transcription was not modified during entrance into stationary phase (one-day-old mycelium contains as much *PaPro1* mRNA as three-day-old one). Its transcription was however slightly increased during perithecium maturation peaking about 24 h after fertilization and decreasing later on (F. Bidard and V. Berteaux-Lecellier, personal communication). Importantly, *PaPro1* transcription was not modified in the *PaMpk1*, *PaMpk2* and *PaNox1* null mutants [38]. Similarly, phosphorylation of PaMpk1 and PaMpk2 was not altered in the *PaPro1* mutants (Figure 3).

### 3.4. Expression of Selected Genes in the PaPro1^Δ^ Mutant

**Figure 4:**
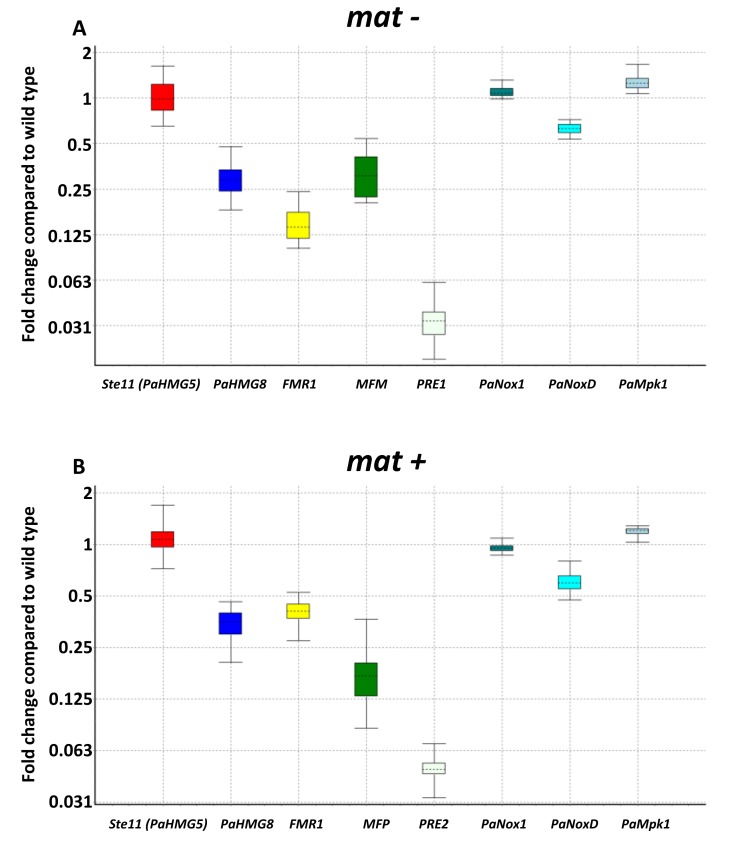
RT-qPCR expression of selected genes in ∆*pro1* mutants. (**A**) *PaPro1*^∆^ *mat−* versus wild type *mat−.* Normalization genes selected with geNorm are *CIT1*, *GPD* and *TBP*, with an average stability value M = 0.08 and V_3/4_ = 0.025 (Table_SI_3_geNorm). (**B**) *PaPro1*^∆^*mat+* versus wild type *mat+*. Normalization genes selected with geNorm are *CIT1*, *GPD* and *TBP*, with an average stability value M = 0.10 and V_3/4_ = 0.024 (Table_SI_3_geNorm). Expression ratios of the following genes have a *p*-value below 1% and their CI 95% does not include 1: *HMG8*, mating-type genes (*FMR1*, *FPR1*), pre-propheromone genes (*MFM*, *MFP*), pheromone-receptor genes (*PRE1*, *PRE2*), *PaNoxD* and *PaMpk1*. In contrast, *STE11* and *PaNox1* did not show any statistically significant difference of expression ratio in any conditions.

In *S. macrospora*, *Pro1* has been shown to regulate many genes of the mak1(=Mpk1)/Nox1 pathway [39,40,41]. We have thus tested by RT-qPCR whether *PaPro1* also regulated orthologous genes from this pathway by comparing the expression of PaMpk1, PaNox1 and PaNoxD in 4-day-old mycelium, i.e., at a time point when wild-type mycelium was competent for fertilization [25]. We found that expression of PaNoxD was reduced by a factor two in the *PaPro1***^Δ^** mutant and expression of PaMpk1 was induced by a factor 1.2, while expression of PaNox1 was not affected (Figure 4).

As stated above, the *PaPro1***^Δ^** mutant appears to have a defect in the production of the zygotic tissues, possibly by lack of fertilization. We thus tested whether the transcription of genes important for fertilization were altered in *PaPro1***^Δ^**. In *P. anserina*, fertilization is controlled by pheromones and receptors (MFM/PR1 in the *mat−* and MFP/PRE2 in the *mat+*), whose expression is under the control of the mating-type-specific transcription factors FPR1 in the *mat+* strain and FMR1 in the *mat−* strain. In turn, the *FPR1* and *FMR1* genes are under the direct control of two HMG transcription factors Ste11 (=PaHMG5) and PaHMG8 [26]. As seen on Figure 4, PaPro1 regulates PaHMG8 and not Ste11, because only transcripts from PaHGM8 were downregulated in *PaPro1***^Δ^**. Transcripts of the mating-type transcription factor*FMR1*, the *mat−* mating pheromone gene *MFM* and receptor gene *PRE1* were also downregulated in the *mat−* strain and the mating-type transcription factor *FPR1*, the *mat+* pheromone gene *MFP* and receptor gene *PRE2* in the *mat+* strain, in accordance with the proposed model of mating type regulation in *P. anserina* [25].

To assess whether PaPro1 may act by directly binding the promoters of these genes, we searched for the presence for the GGCGCTTA binding motif defined for the *S. macrospora* Pro1 protein [39] in their upstream sequences using FIMO [42]. Pro1 motifs were found with a *p*-value < 10^−4^ in the intergenic upstream regions of *PaHMG8*, *FPR1*, *PaNox1* and *PaMpk1* (Table 1). We could not detect them with this probability in the one of *PaNoxD*; however, 10 motifs were found in the intergenic region upstream of *PaNoxD* with a *p*-value < 0.001, including 3 within the first Kb upstream of the translation start site. Note that none were present close to the *Ste11* transcription start site, although two motifs could be detected farther away in the intergenic region upstream of *Ste11* with a *p*-value < 0.001. This suggested that *PaPro1* may regulate directly *PaHMG8*, *FPR1*, *PaMpk1* and possibly *PaNoxD*. It may also regulate directly *PaNox1* but in conditions not investigated here since we could not detect any effect of the deletion of *PaPro1* on *PaNox1* expression although a putative binding site is present in the intergenic region upstream of *PaNox1*. For the other genes, regulation may be direct and/or indirect through *PaHMG8* for *MFM*, *PRE1* and *PRE2* since putative Pro1 binding motifs are found in the promoters of these genes, while regulation is likely solely indirect for *FMR1* and *MFP* since we could not detect Pro1 binding motifs with FIMO.

### 3.5. Inactivation of IDC4, a Gene Coding for a Protein with an AIM24 Domain, Creates an IDC Phenotype

As for *PaPro1*, to prove that inactivation of *IDC4* was indeed responsible for the IDC phenotype, we deleted the *IDC4* gene using the split marker method (see Section 2) and verified the deletion by Southern blot analysis (Figure S1). The *IDC4* deletion (*IDC4*^Δ^) created a typical “pink” IDC phenotype (Figure 1). Further evidence of *IDC4* being the actual gene involved in the IDC phenotype came from mapping analysis that showed that this mutation was located at the centromere of chromosome 2 [2], the position occupied by IDC4. Final proof came from the introduction of a wild-type allele that restored a wild-type phenotype both in *IDC*^508^ and *IDC4*^Δ^ (Figure 1).

**Figure 5:**
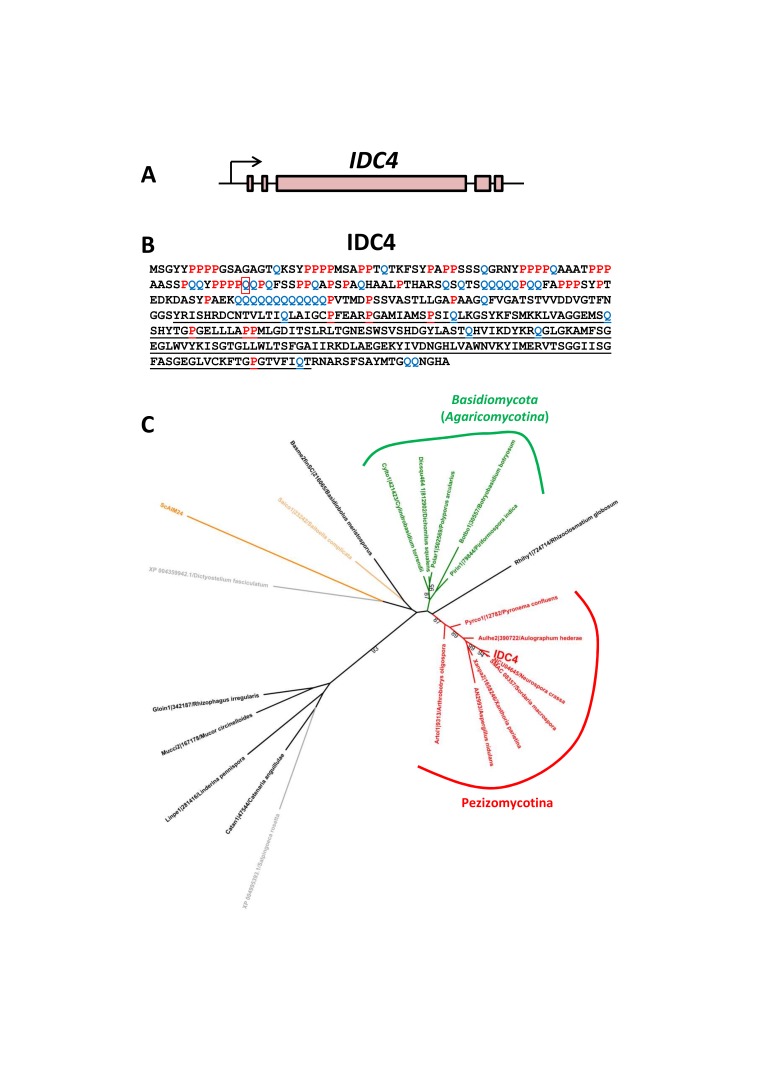
(**A**) Structure of the *IDC4* gene: red boxes are exons. (**B**) Structure of the IDC4 protein. The AIM24 domain is underlined. The prolines are in red and the glutamines in blue. The position of the glutamine codon changed into a stop codon in *IDC*^508^ is boxed. (**C**) Phylogenetic tree constructed with selected IDC4 paralogues and/or orthologues.

*IDC4* has no paralogue in the *P. anserina* genome and encodes a 399-amino-acid-long protein with a predicted molecular weight of 42.5 kD. Expressed Sequence Tags [19] validated the presence of four introns (Figure 5A). Domain analysis identified an AIM24-like domain (=Domain of Unknown Function DUF124) at the C-terminus of the protein (Figure 5B). This domain is present in many proteins from the three domains of life, i.e., *Eukaryota*, *Archaea* and *Eubacteria*. The N-terminus of the IDC4 protein is rich in proline (there are 36 prolines in the first 120 amino acids) and glutamines (e.g., two poly-glutamine stretches are present at position 99–111 and 132–143) (Figure 5B). Exploration of the genome sequences of fungi by BLAST at Mycocosm (http://genome.jgi.doe.gov/programs/fungi/index.jsf) identified potential orthologues in the genome of many but not all fungi. *IDC4* orthologues and/or paralogues genes seem present in a patchy distribution in both the *Dikarya* and basal fungi. Most species of *Ascomycota* have at least one copy, while most *Basidiomycota* appear to lack an *IDC4* orthologue and/or paralogue. Especially, *Ustilaginomycotina* and *Puccinomycotina* seem to be devoid of *IDC4* orthologues. The proline and glutamine-rich N-terminus region is conserved in the orthologous proteins from the *Pezizomycotina* and to a lesser extend in those from the *Taphrinomycotina* and some *Basidiomycota* (Figure S5). Such region is absent in the other proteins carrying an AIM24 domain from *Bacteria*, *Archea* and *Eukaryota*, including the ScAIM24 protein from *Saccharomyces cerevisiae*. The *N. crassa* orthologue of *IDC4* (NCU04645) has been recently demonstrated to be involved in anastomosis [43]. The only *S. cerevisiae* protein with an AIM24 domain is the ScAIM24 protein. However, ScAIM24 seems distantly related to IDC4 and lacks the proline and glutamine rich N-terminus (Figure 5C and Figure S5). It has instead a mitochondrial targeting sequence that directs ScAIM24 into the mitochondria, where it participates to the integrity of the “Mitochondrial contact site and Cristae Organizing System” (MICOS) [44]. We did not detect any mitochondrial targeting signal in IDC4 nor in the proteins from the *Pezizomycotina* and *Basidiomycota* that we tested, using MitoFates (http://mitf.cbrc.jp/MitoFates/cgi-bin/top.cgi) and MitoProtII (https://ihg.gsf.de/ihg/mitoprot.html). Phylogenetic analysis suggested that ScAIM24 was likely paralogous to IDC4 rather than orthologous. However, statistical support for paralogy was weak (Figure 5C).

### 3.6. Fine Analysis of the IDC Phenotype of the IDC4 Mutants

Both *IDC*^508^ and *IDC4*^Δ^ had the same typical IDC phenotype (Figure 1 and Figure S2), suggesting that the stop codon early in the *IDC4* reading frame in *IDC*^508^ created a null allele. Both mutants were able to differentiate ascogonia and spermatia. Grafting analysis showed that the gene was important in the mycelium, because two-day-old wild-type perithecia grafted onto mycelia of the IDC4 mutants did not continue their development (Figure 2A). It was dispensable in the maturing fruiting bodies, because *IDC4*^Δ^ × *pks1-193* and *IDC*^508^ × *pks1-193* mosaics of compatible mating types differentiated numerous darkly pigmented perithecia and *IDC4*^Δ^ *mat+* × *IDC4*^Δ^ *mat−* × Δ*mat* and *IDC*^508^ *mat+* × *IDC*^508^ *mat−* × Δ*mat* trikaryons produced many fruiting bodies that matured like wild-type ones and generated abundant progeny. Finally, fertility of the IDC4 mutants was not restored by providing paper in the growth medium (Figure S3) and the *IDC4* mutants were able to differentiate the appressorium-like structures permitting cellophane penetration.

### 3.7. Expression of IDC4

Analysis of microarray data [38] and (F. Bidard and V. Berteaux-Lecellier, personal communication), indicated that transcription of *IDC4* was not regulated during mycelium growth and perithecium development. *IDC4* transcription was also not affected in the PaMpk1, PaNox1 and PaMpk2 mutants. Also, phosphorylation of PaMpk1 and PaMpk2 was not altered in the mutants (Figure 3).

### 3.8. IDC4 is Localized in the Cytosol

**Figure 6:**
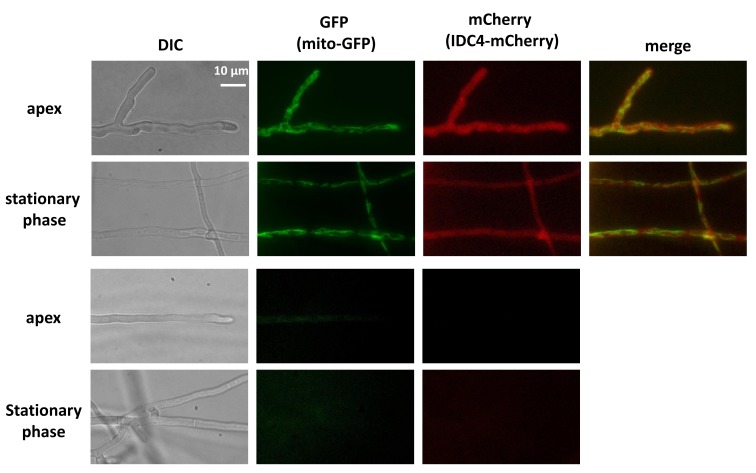
Cellular localization of IDC4. The two top rows are the strain carrying the mito-GFP and IDC4-mCherry transgenes. Red fluorescence in apical and stationary phase hyphae from 3-day-old hyphae carrying both transgenes was located in the cytosol, while the green fluorescence was detected in elongated mitochondria. The two bottom rows are from the wild type that does not carry transgenes; no fluorescence was detected in the wild type.

To determine the cellular localization of the IDC4 protein, it was tagged at its N-terminus with the mCherry protein. To this end, a circular DNA fragment eliminating the *IDC4* stop codon and carrying in frame the mCherry partial CDS was inserted at the *IDC4* locus (see Section 2). This permitted to produce an IDC4-mCherry fusion protein under the control of the *IDC4* promoter. A thallus containing a functional transgene expressing the fusion protein was crossed with a strain carrying the mito-GFP transgene [24]. In the progeny, we recovered strains carrying both transgenes, enabling to visualize at the same time IDC4 and mitochondria (Figure 6). IDC4-mCherry was located in the cytosol and not the mitochondria, as expected from the lack of mitochondrial targeting signal.

### 3.9. Double Mutant Analyses Enable to Place PaPro1 and IDC4 Upstream of PDC1

**Figure 7:**
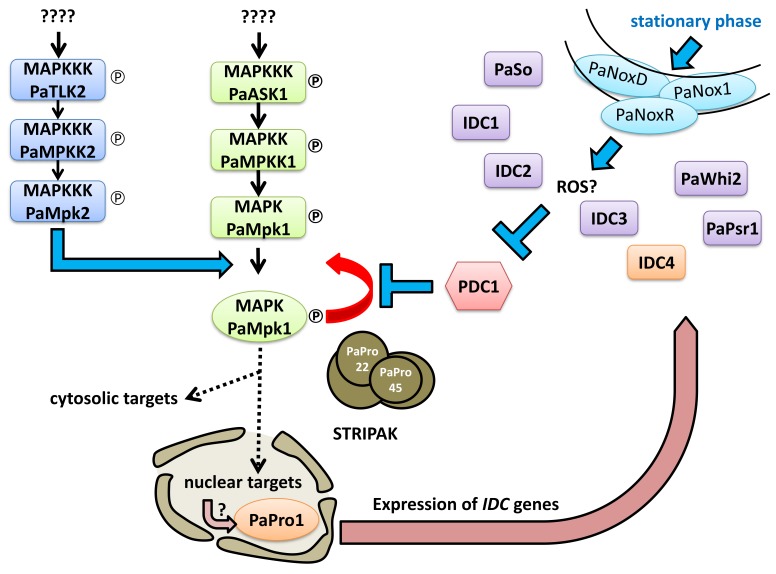
Scheme of the IDC signaling cascade. PDC1 encodes a repressor of the cascade, which was shown to likely repress the positive regulatory loop of the PaMpk1 MAPK cascade [45]. Double mutants of *PDC1* and either *PaMpk1* or *PaMpk2* never present CG. On the contrary, double mutants lacking PDC1 and either one of the upstream IDC proteins may exhibit CG sectors during growth since the positive regulatory loop is not repressed, yet these are not induced by passage into stationary phase, because the signal between the PaNox1 complex and the MAPK is not transmitted.

**Figure 8:**
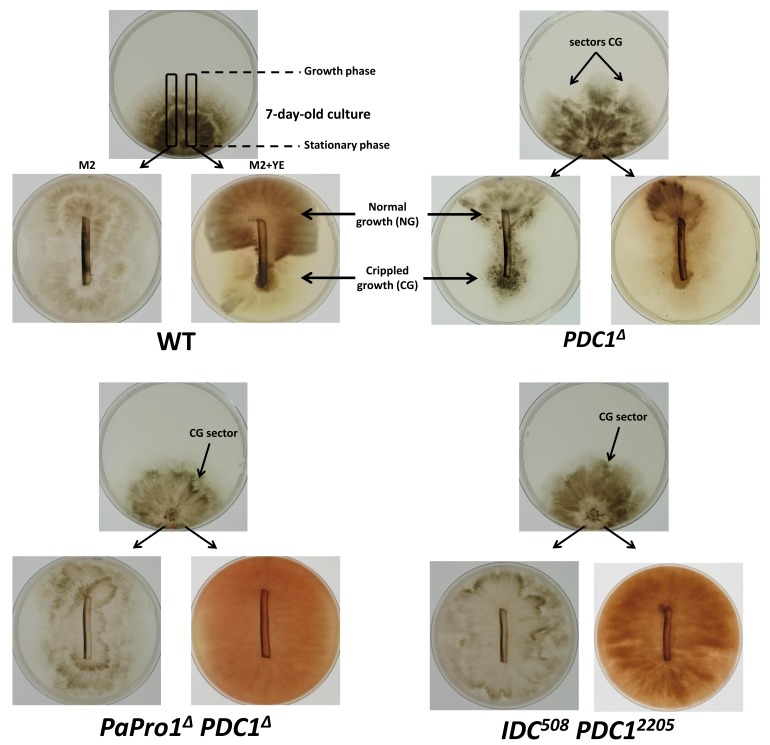
Epistasis analysis with *PDC1* mutants. CG test were made as in Figure 1, i.e., slices were taken on seven-day-old mycelia with the indicated genotypes and reinoculated either on M2 or on M2 containing YE. Pictures were taken seven days later. For the wild-type this resulted in the development of CG only on medium containing YE, whereas for the *PDC1*^Δ^ mutant, CG developed on both media. The *PaPro1*^Δ^ *PDC1*^Δ^ and *IDC*^508^ *PDC*^2205^ double mutants exhibited spontaneous CG sectors on M2, but could not be induced to present CG after passage into stationary phase.

In previous analyses [2,45], we positioned IDC proteins with respect to PDC1 by double mutant analysis. PDC1 encodes a repressor within the PaMpk1/PaNox1 pathway and is positioned between the PaMpk1/PaMpk2 MAP kinases and the other members of the cascade (Figure 7). To position PaPro1 and IDC4 within the cascade, we constructed the *PaPro1*^Δ^ *PDC1*^Δ^ and *IDC*^508^ *PDC*^2205^ double mutants; *PDC*^2205^ is a null allele of *PDC1* [45]. Phenotypic analyses of these mutants (Figure 8) showed that both lacked pigments, aerial hyphae and were female sterile, yet both developed CG sector on M2. Moreover, CG was not inducible by stationary phase in both double mutants (Figure 8). Overall, these suggested that PaPro1 and IDC4 were positioned upstream of PDC1 in the cascade (Figure 7). Indeed, it appeared (1) that the stationary phase signal triggering CG was blocked and (2) that the positive feedback loop enabling CG was still functional and removal of *PDC1* lifted the repression on this loop in both mutants.

### 3.10. Mutants of the STRIPAK Complex Do Not Have a Typical “Pink” IDC Phenotype

Both in *N. crassa* and *S. macrospora*, the orthologues of the PaMpk1 and PaMpk2 MAPK cascades and the PaNox1 Nox are known to act along with components of the striatin-interacting phosphatases and kinases (STRIPAK) complex (see [46] for a review). This complex is conserved during evolution in eukaryotes and is involved in the regulation of many cellular processes including anastomoses and sexual development in *N. crassa* and *S. macrospora*. Intriguingly, none of the “pink” *IDC* mutant that we have identified was affected in a gene encoding component of STRIPAK. To evaluate the phenotype of STRIPAK mutants in *P. anserina*, we deleted the orthologues of *S. macrospora pro22* (=*Pa_2_9440* or *PaPro22*) and *pro45* (=*Pa_1_15490* or *PaPro45*) [47,48]. These are known in *N. crassa* as *Ham-2* and *Ham-5*, respectively [49]. In both cases, the coding sequences were replaced by a hygromycin B resistance marker (see Section 2).

**Figure 9:**
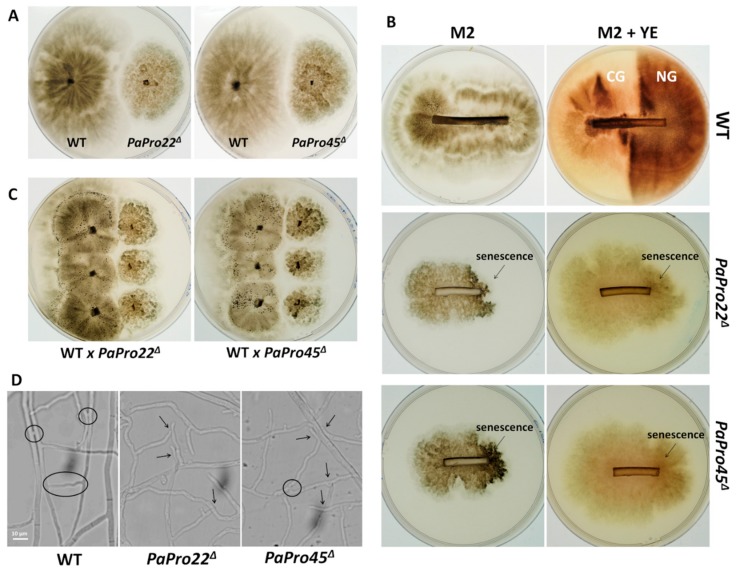
Phenotypes of *PaPro22*^Δ^ and *Papro45*^Δ^**.** (**A**) The wild type (WT), *PaPro22*^Δ^ and *Papro45*^Δ^ were inoculated at the same time on M2 plates and the pictures were taken ten days later. *PaPro22*^Δ^ and *Papro45*^Δ^ have a slow growth, are devoid of aerial hyphae, but accumulate pigments. (**B**) CG test were made as those of Figure 1 and Figure 7. Apical hyphae of *PaPro22*^Δ^ and *Papro45*^Δ^ show after 3–4 cm the arrest of growth and accumulation of dark pigments typical of senescence. (**C**) Sexually compatible mycelia of the indicated genotypes were inoculated on M2 and three days later 2 mL of water were added to spread spermatia. The wild-type thalli differentiated after seven days mature perithecia, but not the mutant ones, showing that *PaPro22*^Δ^ and *Papro45*^Δ^ were ♀-sterile and ♂-fertile. Perithecia are the small black dots. (**D**) Anastomoses (open circle) are frequent in the wild-type, but very rare in *PaPro22*^Δ^ and *Papro45*^Δ^ (a field in which one figure of anastomose was seen is shown for the *Papro45*^Δ^ mutant, in most fields anastomoses could not be observed). Hyphae from *PaPro22*^Δ^ and *Papro45*^Δ^ often meet, but do not engage cell fusion (arrows).

**Figure 10:**
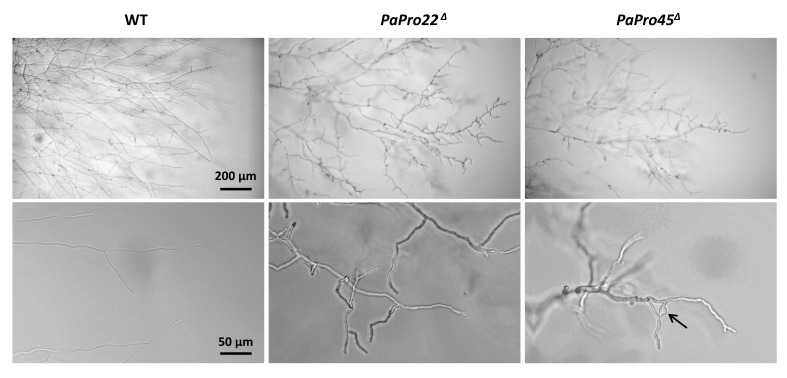
Morphology of WT and STRIPAK mutant hyphae. Pictures were taken with objectives ×10 (**top**) and ×40 (**bottom**). Arrow points towards inflated hyphae.

The *PaPro22*^Δ^ *and PaPro45*^Δ^ deletion mutants had the same phenotype (Figure 9). Their mycelium had a characteristic slow growing wavy mycelium (speed = 4.5 mm/d; wild type speed = 7.5 mm/d; Figure 9A). It lacked aerial hyphae, but accumulated pigments like the wild type (Figure 9A). Both mutants lacked CG, but exhibited rapidly senescence (Figure 9B). They were male-fertile and female sterile (Figure 9C). Microscopically, *PaPro22*^Δ^ *and PaPro45*^Δ^ mycelia were abnormal (Figure 10). Hyphae were thicker (width= 6.5 ± 0.5 µm, wild type: 4.8 ± 0.3 µm), some even inflated. They frequently exhibited orientation changes giving them a wavy morphology. The regular branching of the wild-type hyphae was replaced by short stunted branches, themselves often carrying additional small branches. These alterations in the morphology of the hyphae accounted for the slow growth of both mutants. Anastomoses were severely reduced, but not completely abolished in *PaPro22*^Δ^ and *PaPro45*^Δ^ (Figure 9D), as seen in *Epichloe festucae* for a mutant of the STRIPAK complex affected in *MobC* [50]. To assess whether *PaPro22*^Δ^ and *PaPro45*^Δ^ were proficient in engaging cell fusion with wild-type hyphae, the *PaPro22*^Δ^ and *PaPro45*^Δ^ hygromycin B-resistant mycelia were mixed with geneticin-resistant mycelia—otherwise like the wild type in their ability to engage anastomoses—and inoculated on M2 medium to allow for cell fusion. Mycelium plugs were then replicated onto medium containing both hygromycin B and geneticin. None of the tested plugs yielded mycelia resistant to both antifungal substances, indicating that *PaPro22*^Δ^ and *PaPro45*^Δ^ were not able to efficiently engage anastomoses with the wild type.

Because *PaPro22*^Δ^ and *PaPro45*^Δ^ could not engage anastomoses including with the wild type, we could not test whether they did or did not express the C element during stationary phase and hence had a phenotype similar to the “pink” IDC mutants or the other “non-pink” ones. We however constructed *PaPro22*^Δ^ *PDC1*^Δ^ and *PaPro45*^Δ^ *PDC1*^Δ^ double mutants, to localize their position with respect to the IDC pathway. Both double mutants never presented CG sectors, although this was difficult to definitively ascertain because they presented senescence rapidly (Figure S6). Nevertheless, CG could not be induced in *PaPro22*^Δ^ *PDC1*^Δ^ and *PaPro45*^Δ^ *PDC1*^Δ^ by incubation in stationary phase (Figure S6), suggesting that *PaPro22*^Δ^ and *PaPro45*^Δ^ were downstream in the cascade (Figure 7).

**Figure 11:**
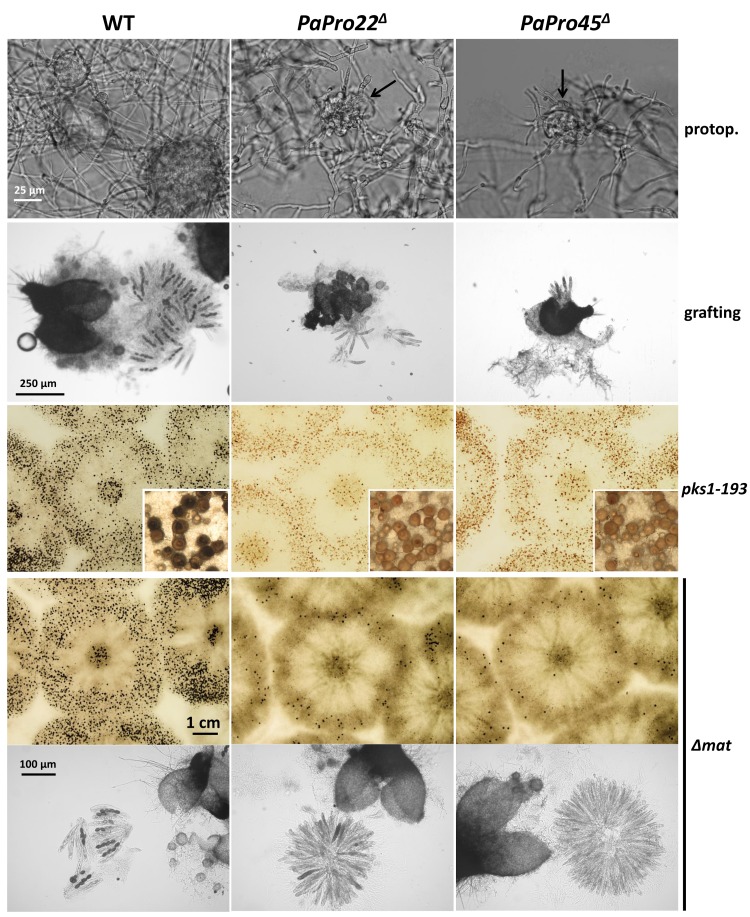
Fertility defect of the *PaPro22*^Δ^ and *Papro45*^Δ^ mutants. From top to bottom: Both *PaPro22*^Δ^ and *Papro45*^Δ^ differentiate small and abnormal-looking protoperithecia (arrows). Wild type perithecia grafted onto *PaPro22*^Δ^ and *Papro45*^Δ^ mycelia stopped their development, while those grafted onto wild type (WT) continued their development by enlarging and producing abundant progeny. Heterokaryons between psk1-193 and the *PaPro22*^Δ^ and *Papro45*^Δ^ mutants produced only non-pigmented fruiting bodies, while those made with the wild-type produced both pigmented and non-pigmented fruiting bodies. Trikaryons between *PaPro22*^Δ^ and *Papro45*^Δ^ produced few perithecia, unlike the wild type. These did not contain mature asci; observation of the rosette of asci in the mutants showed that their maturation was blocked before ascospore delimitation.

The female fertility defect of *PaPro22*^Δ^ and *PaPro45*^Δ^ was analyzed in depth as for the *PaPro1* and *IDC4* mutants. We were able to observe spermatia and protoperithecia on one-week-old *PaPro22*^Δ^ and *PaPro45*^Δ^ mycelia. Protoperithecia were however much smaller than those differentiated by the wild type and appeared abnormally shaped (Figure 11). Wild-type perithecia grafted onto *PaPro22*^Δ^ and *PaPro45*^Δ^ mycelia stopped their development (Figure 11), showing that *PaPro22* and *PaPro45* were required in the mycelium for fruiting body maturation. We then performed mosaic analyses with *psk1-193*, the mutation creating a cell-autonomous pigmentation defect that can be used to trace the origin of the maternal tissues of the fruiting body [35,51]. Data showed that fertilization-competent *pks1-193*/*PaPro22*^Δ^ and *pks1-193*/*PaPro45*^Δ^ heterokaryons produced only non-pigmented fruiting bodies, indicated that *PaPro22* and *PaPro45* were required in the developing perithecia (Figure 11). Mosaic analyses with Δ*mat* showed that the trikaryons with *PaPro22*^Δ^ and *PaPro45*^Δ^ were able to produce few fruiting bodies. These at first glance appear mature, yet when the content of these perithecia was analyzed, the rosettes of asci were found to be blocked at an early stage of ascus maturation, before the delimitation of the ascospores (Figure 11). *PaPro22* and *PaPro45* were thus required for the maturation of the zygotic tissues. Note that, owing to the severe growth impairment of *PaPro22*^Δ^ *and PaPro45*^Δ^, the recovery of small numbers of fruiting bodies was expected since fertilization-competent *PaPro22*^Δ^ and *PaPro45*^Δ^ hyphae are likely in small amounts in the trikaryons. Hence, the STRIPAK complex is necessary in the mycelium, the maternal tissues and the zygotic tissues, to enable fruiting body development.

## 4. Discussion

Crippled Growth is an unusual degenerative process that likely results from the abnormal activation of a MAP kinase cascade. It is a model to understand how regulatory networks can create epigenetic inheritance [52]. Through complete genome sequencing and targeted gene inactivation, we have identified two genes, *PaPro1* and *IDC4*, involved in the development of CG and other important features of *P. anserina* life including differentiation of structures typical of stationary phase (anastomoses) and sexual reproduction (perithecia). We have also investigated the role of the STRIPAK complex in CG and other stages of the life cycle of *P. anserina*, by analyzing mutants deleted for the orthologues of *S. macrospora pro22* and *pro45* [47,48]. 

Orthologues of *PaPro1* have previously been investigated in *N. crassa* [49,53], *E. festucae* [54,55], *Alternaria brassicola* [56], *Cryphonectria parasitica* [57], *Fusarium graminearum* [58], *Aspergillus nidulans* [59,60], *Aspergillus fumigatus* [61], and especially in *S. macrospora*, in which this transcription factor was discovered [33,34,39,40,41]. These studies identified target genes regulated by this transcription factor, including orthologues of some of the pink *IDC* genes (Table 2) and among them orthologues of *PaNoxD*, *PaNox1* and *PaMpk1* [39,40,41,54,55,57]. RT-qPCR showed that *PaNoxD* and *PaMpk1* transcripts were down- and up-regulated, respectively in the PaPro1^Δ^ mutant, while *PaNox1* transcription level was not significantly changed. Additionally, analyses of the microarrays from [38] showed that *PaPro1* transcription was not modified in the *PaNox1*, *PaMpk1* and *PaMpk2* mutants. Based on these data, we propose that *PaPro1* is a regulator of the expression of the *IDC* genes (Figure 7). This is confirmed by the *PaPro1*^Δ^ *PDC1*^Δ^ double mutant analysis that indicated that PaPro1 acts likely upstream of the MAPK cascades. However, owing to the postulated self-regulatory loop present in this cascade [4], it remains to be determined whether PaPro1 is a phosphorylation target of the PaMpk1/PaMpk2 MAP kinases. Based on the phenotype of the IDC mutants, we previously hypothesized [4,5] that the PaPro1-containing signaling pathway controls entrance into stationary phase, i.e., the nutrient starvation response, a feature also more recently found in *E. festucae* for its orthologue *ProA* [55].

Another role of *PaPro1* is the activation of the sexual recognition pathway, including *PaHMG8*, the mating type and the pheromone and receptor genes. Surprisingly, in *S. macrospora*, the pheromone genes *ppg1* and *ppg2* were found to be up-regulated in sexually competent mycelia and protoperithecia mutant for *pro1* [40,62]. This species is homothallic and does not require fertilization between a male and a female gamete for sexual reproduction, a feature possibly accounting for the different regulation of the pheromone genes by *PaPro1* and *S. macrospora pro1*. In *E. festucae*, a RNA seq analysis evidenced a regulation in the same sense as in *P. anserine*, since the pheromone receptor gene *pre-1* was found to be activated by ProA [55]. Previous work in *P. anserina* demonstrated that two HMG-box transcription factors, STE11 and PaHMG8, control the expression of mating-type genes at the stage of fertilization [26]. Transcription of STE11 is not affected by PaPro1, suggesting that PaHMG8 specifically mediates the action of *PaPro1*. We propose that PaPro1 connects the nutrient status of the mycelium with its sexual competency through HMG8, i.e., it enables fertilization only when the mycelium is starved. Note that differentiation of the gametes is independent of PaPro1 but also of the mating type, the pheromone and receptor genes as well as the HMG genes that control their expression. At the present time, the signaling pathways enabling male and female gamete formation are unknown in *P. anserina*. Interestingly, once fertilization is achieved the role of *PaPro1* is dispensable in the zygotic tissues, while its presence is mandatory both in the mycelium and the maternal tissues of the fruiting bodies. 

The *IDC4* gene is widely conserved in higher fungi and possesses an AIM24 domain, which is present in proteins from all domains of life. The orthologue in *N. crassa* has been shown to control anastomoses as *IDC4* does [43]. The *S. cerevisiae* gene *AIM24* encodes a mitochondrial protein involved in scaffolding other proteins [44,63]. Based on phylogenetic analysis, it is probably a paralogue rather that an orthologue of *IDC4*. In line with this finding, IDC4 lacks a mitochondrial targeting signal and accordingly is localized in the cytosol, where it could be involved in the scaffolding of some IDC proteins. Analysis of the *IDC*^508^ *PDC*^2205^ double mutant indicates that its position within the cascade is upstream of PDC1. 

With the identification of *PaPro1* and *IDC4*, all the genes mutated in the available pink IDC mutants are now documented (Table 2). The analysis of these mutants recovered by forward genetic analyses of CG has shown that the IDC proteins are all involved in signaling in *P. anserina*. They ensure functions that are conserved in a wide range of fungi, especially in the *Pezizomycotina* in which they have been shown to control anastomosis, which is mostly studied in *N. crassa* (reviewed in [64]), and fruiting body development, mostly analyzed in *S. macrospora* (reviewed in [65]). They also control interactions with hosts in phytopathogens (see for a review [66]), entomopathogens (e.g., [67]) and plant mutualistic fungi (e.g., [54,68,69]). Especially, some *IDC* genes, but not all, are involved in the production of functional appressorium and appressorium-like structures [70,71]. Because in both *N. crassa* and *S. macrospora*, the orthologues of the *IDC* genes work along those encoding for the STRIPAK complex [64,72,73], we deleted the *P. anserina* orthologues of *pro22* and *pro45*, encoding two different subunits of the STRIPAK complex, to assess their role in CG and more generally in *P. anserina* development. Both mutants had features closely resembling those of typical “pink” *IDC* mutants: lack of CG, lack of aerial hyphae and anastomosis, female sterility, while retaining the ability to differentiate female gametangia. They however differed from typical “pink” IDC mutants by having abnormally shaped hyphae, being slow-growing, short-lived, pigmented and blocked at an early stage during the development of the zygotic tissues. Slow growth of the STRIPAK mutant is also observed in *S. macrospora* [73] and *N. crassa* [74]. This indicates that in *P. anserina* the STRIPAK complex genes have additional roles when compared to those of the typical “pink” IDC genes. Owing to their lack of anastomosis, including when confronted with the wild-type, we could not easily assess whether they do or do not make the C element, a problem that we previously encountered with mutants of the PaMpk2 pathway [12]. Whatever their actual role in CG, their slow-growing and short-lived phenotypes likely prevented their retention during the genetic screens performed to select for IDC mutants [2].

**Figure 12:**
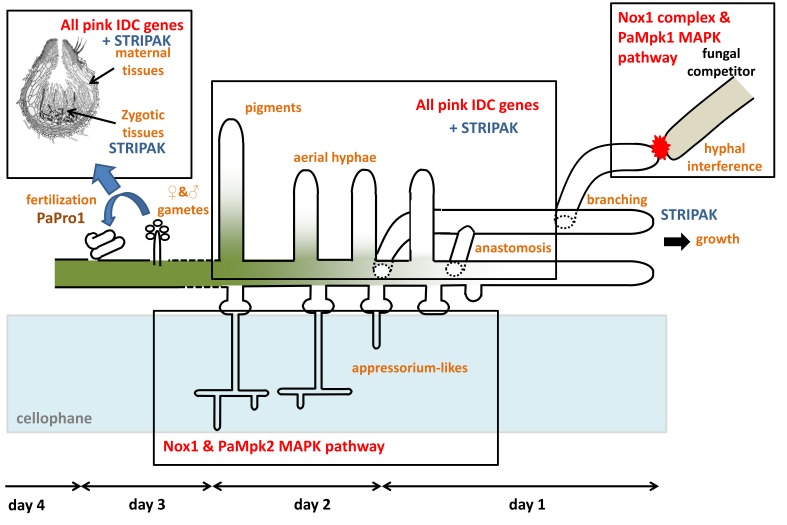
Scheme of the different developmental stages controlled by the *IDC* genes in *P. anserina*.

In *P. anserina*, *IDC* genes have multiple roles (Figure 12). Firstly, they all regulate entry into stationary phase since their absence results in lack of melanin, aerial hyphae and anastomoses. They on the contrary do not seem to regulate apical growth, because all the mutants have a near wild type apical extension speed, and they have a minor role in branching. They also do not regulate the differentiation of male and female gametes. They resume a role during the development of the fruiting bodies either in the mycelium, the peridium of the fruiting body or in both (Table 2). Apart from *PaPro1*, they are not involved in the formation of the zygotic tissue (i.e., in fertilization) and none, including *PaPro1*, appears required in further development of the zygotic tissues, i.e., the production of ascogenous hyphae and the maturation of asci. The fertility of mutants for four of them can be restored by providing paper as food source (Table 2). In the case of The *PaNox1* and *PaNoxD* mutants, it has previously been shown that the PaNox2 Nox complex can take over the PaNox1 one [5]. In the case of *IDC2* and *IDC3*, leakiness of the mutants indicated that additional parallel pathways may take over [10]. Some of these proteins, but not all of them, are required for the production of appressorium-like structures and hyphal interference (Table 2 and Figure 12), suggesting that they may be part of different cascades regulating these two phenomena. For example, PaNoxR regulates not only Nox1, but also Nox2, which is involved in ascospore germination (hence the additional germination defect of the PaNoxR mutants) and appressorium-like formation, but at a stage earlier than PaNox1 [5,9]. Based on the role of the IDC genes, we proposed a model for CG, in which activation of the cascade is able to self-perpetuate thanks to a positive auto-regulatory loop in the PaMpk1 MAPK pathway (Figure 7) [4,6]. The results presented further confirm this model, which should now be further tested by identifying additional genes mutated in the PDC mutants.

## Figures and Tables

**Table 1:** Pro1 motifs found using FIMO analysis in the promoters of selected genes.

Genes	Promoter Size	*p* = 0.001	*p* = 0.0001
*Ste11*	3216	2 (0)	0
*PaHGM8*	1506	4 (2)	2 (1)
*FMR1*	480	0	0
*MFM*	1974	1 (1)	0
*PRE1*	1054	5 (5)	0
*FPR1*	3945	4 (4)	1 (1)
*MFP*	1011	0	0
*PRE2*	1236	1 (1)	0
*PaNoxD*	3774	12 (3)	0
*PaNox1*	3630	10 (3)	3 (1)
*PaMpk1*	1704	5 (3)	1 (1)

In parentheses are the numbers of motifs found when 1 kb upstream the translation start site of the genes are analyzed.

**Table 2:** Genes identified by the analysis of pink IDC mutants.

Gene	Id from the Genome Project	Available Mutants	Function	Ref.	*N. crassa* (*S. macrospora*) Orthologue	Site of Action during Perithecium Development	Hyphal Interference	Appressorium-Like	Fertility on Paper	Additional Phenotype
*PaASK1*	*Pa_5_9370*	*IDC*^118^, *IDC*^172^, *IDC*^507^	MAPKKK	[4]	mik1 (mik1)	mycelium	−	+	−	
*PaMKK1*	*Pa_7_10270*	*IDC*^404^, *IDC*^505^	MAPKK	[6]	mek-1 (mek1)	mycelium	−	+	−	
*PaMpk1*	*Pa_2_13340*	Δ*PaMpk1*	MAPK	[6]	mak-1 (mak1)	mycelium	−	+	−	
*PaSo*	*Pa_1_7440*	*IDC*^821^, *PaSo*^Δ^	MAPK1 scaffold?	[7]	So = Ham-1 (pro40)	peridium	+/−	+	−	
*PaTLK2*	*Pa_7_8030*	*IDC*^510^, Δ*PaTLK2*	MAPKKK	[12], this paper	NRC-1	mycelium	+	−	−	no ascospore germination
*PaMKK2*	*Pa_2_820*	Δ*PaMKK2*	MAPKK	[12]	mek-2	mycelium	+	−	−	no ascospore germination
*PaMpk2*	*Pa_5_5680*	Δ*PaMpk2*	MAPK	[12]	mak-2	mycelium	+	−	−	no ascospore germination
*IDC1*	*Pa_3_8520*	*IDC*^1^, *IDC*^318^, *IDC*^502^	MAPK2 scaffold?	[13]	Ham-5	mycelium and peridium	+/−	+	−	
*PaNox1*	*Pa_1_2410*	*IDC* ^343^	Nox catalytic	[5]	nox-1 (nox1)	peridium	+/−	−	+/−	
*PaNoxD*	*Pa_1_7250*	*IDC*^509^, *PaNoxD*^Δ^	Nox docking	[8]	Ham-6 (pro41)	peridium	+/−	−	+/−	
*PaNoxR*	*Pa_7_11300*	*IDC*^524^, Δ*PaNoxR*	Nox regulator	[9]	NOR-1 (nor1)	peridium	+/−	−	-	no ascospore germination
*IDC2*	*Pa_1_16080*	*IDC*^506^, *IDC*^519^, *IDC3*^Δ^	? GPI anchored	[10]	Ham-7	diffusible	+	+	+/−	
*IDC3*	*Pa_1_1990*	*IDC*^522^, *IDC3*^Δ^	? transmembrane	[10]	Not yet studied	diffusible	+	+	+/−	
*IDC4*	*Pa_2_230*	*IDC*^508^, *IDC4*^Δ^	? cytosolic	this paper	NCU04645	mycelium	+/−	+	−	
*PaPsr1*	*Pa_1_3870*	*scle1*, *PaPsr1*^Δ^	?	[11]	psr-1	peridium	+	+	−	
*PaWhi2*	*Pa_4_7330*	*IDC*^815^, *PaWhi2*^Δ^	Phosphatase?	[11]	whi-2	peridium	+	+	−	
*PaPro1*	*Pa_1_10140*	*IDC*^511^, *PaPro1*^Δ^	Transcription factor	this paper	ADV-1 (pro1)	mycelium and peridium (+ fertilization)	+/−	+	−	

+: phenomenon still present in the mutants, −: phenomenon absent in the mutants, +/−: phenomenon diminished in the mutants. ? = uncertain.

## Supplementary Materials

The following are available online at http://www.mdpi.com/2309-608X/4/3/85/s1, Table S1. Primers used; Table S2. Cq from RT-qPCR experiments; Table S3. geNorm analyses of housekeeping genes expression in WT and PaPro1^∆^; Table S4. REST analysis of gene expression in WT and *PaPro1*^∆^; Figure S1. Southern blot analysis of the *PaPro1* and *IDC4* deletion mutants. On the left, schematic representation of the wild-type and deleted loci. Enzymes and position of the probes (double arrows) used for Southern blots are indicated; on the right, corresponding autoradiograms. All tested Mutants had correct gene replacement; Figure S2. Lack of anastomosis in the *IDC4* and *PaPro1* mutants. Many anastomoses (open circles) can be detected in the wild-type mycelium, while none can be in *IDC*^511^ and *IDC*^508^. Arrows points towards contacts between hyphae without cell fusion. The *PaPro1*^Δ^ and *IDC4*^Δ^ mutants had the same defects as *IDC*^511^ and *IDC*^508^; Figure S3. Rescue of fertility by paper. *mat+/mat−* heterokaryotic mycelia of the indicated genotypes were incubated for 10 days on medium containing paper, at which time the pictures were taken. Mutants of *PaPro1* and *IDC4* were not rescued by paper, unlike the *PaNox1* mutant; Figure S4. Hyphal Interference in wild type and mutants. Thallus of *P. anserina* was inoculated at the center and three thalli of *Penicillium chrysogenum* were inoculated 1.5 cm away. After three days of incubation, the peroxide burst typical of hyphal interference was visualized by precipitation of diaminobenzidine (Top, arrow shows the halo of peroxide produced by *P. anserina* at the contact with *P. chrysogenum*) and *P. chrysogenum* cell death (bottom, arrow) by coloration with trypan blue [36]. Both *IDC*^508^ and *IDC*^511^ were impaired in the production of peroxide and the killing of *P. chrysogenum*; Figure S5. Alignment of selected IDC4 orthologues/paralogues. The prolines are highlighted in pink and the glutamines in blue. The AIM24 domain is underlined in red; Figure S6. CG test on in *PaPro22*^Δ^ *PDC1*^Δ^ and *PaPro45*^Δ^ *PDC1*^Δ^ double mutants. Tests were made as described in Figure 1 and Figure 8.
