# Supplementary material for: PaPro1 and IDC4, Two Genes Controlling Stationary Phase, Sexual Development and Cell Degeneration in Podospora anserina
Source: J Fungi (Basel). 2018 Jul 11;4(3):85. doi: 10.3390/jof4030085 (PMC6162560; doi:10.3390/jof4030085)
Supplement: Supplementary file 1 [file jof-04-00085-s001.zip › supplementary figures.pdf]

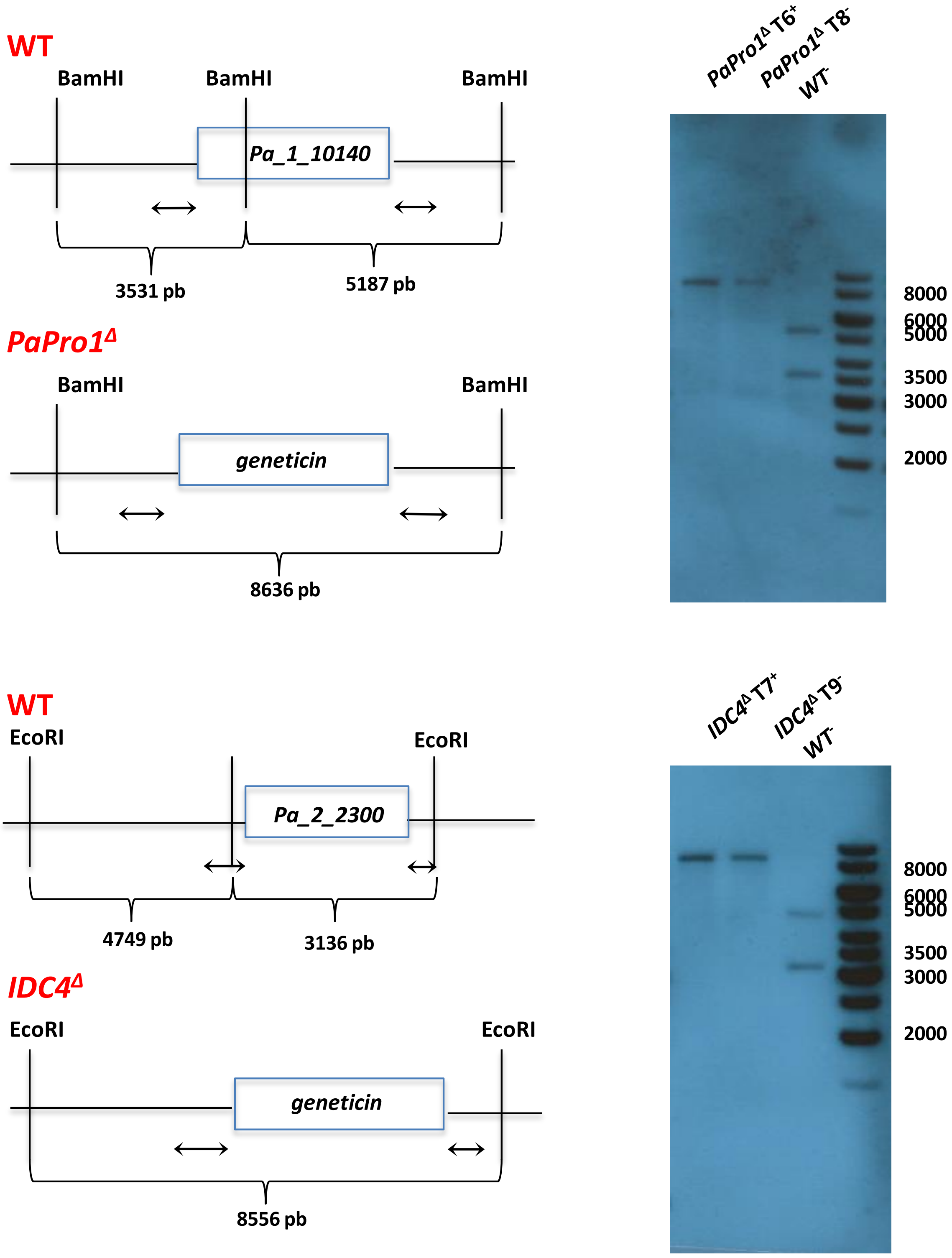

Fig. S1. Southern blot analysis of the *PaPro1* and *IDC4* deletion mutants. On the left, schematic representation of the wild-type and deleted loci. Enzymes and position of the probes (double arrows) used for Southern blots are indicated; on the right, corresponding autoradiograms. All tested Mutants had correct gene replacement.

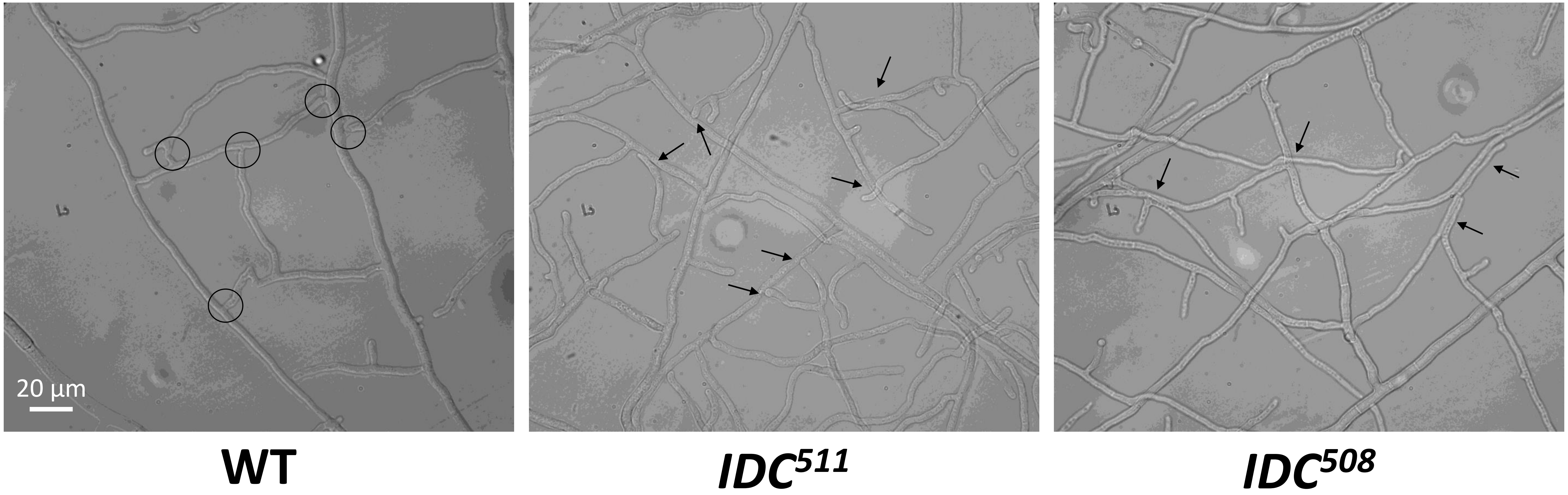

Fig. S2. Lack of anastomosis in the *IDC4* and *PaPro1* mutants. Many anastomoses (open circles) can be detected in the wild-type mycelium, while none can be in *IDC*^{511} and *IDC*^{508}. Arrows points towards contacts between hyphae without cell fusion. The *PaPro1*$^{\Delta}$ and *IDC4*$^{\Delta}$ mutants had the same defects as *IDC*^{511}and *IDC*^{508}.

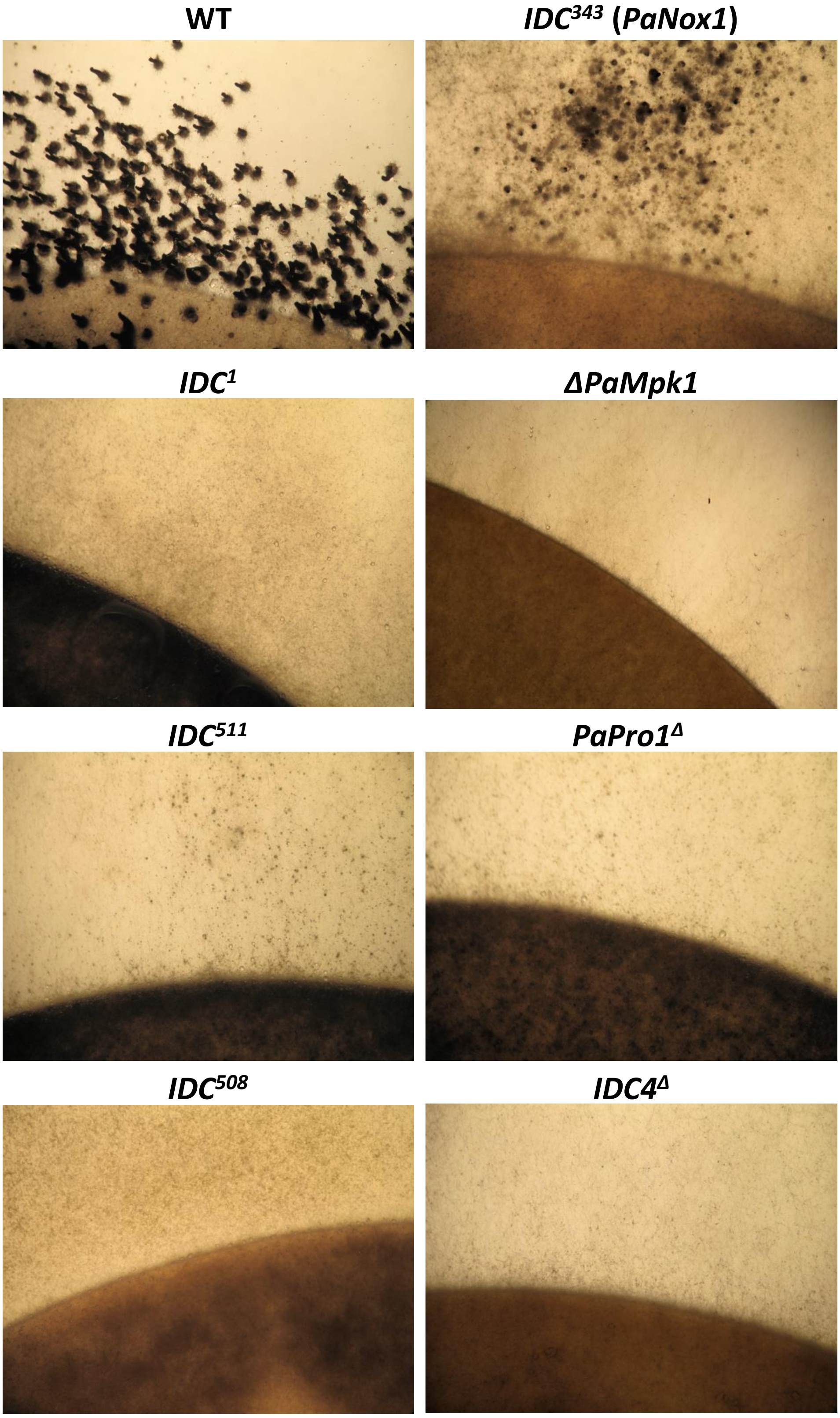

Fig. S3. Rescue of fertility by paper. *mat+/mat-* heterokaryotic mycelia of the indicated genotypes were incubated for 10 days on medium containing paper, at which time the pictures were taken. Mutants of *PaPro1* and *IDC4* were not rescued by paper, unlike the *PaNox1* mutant.

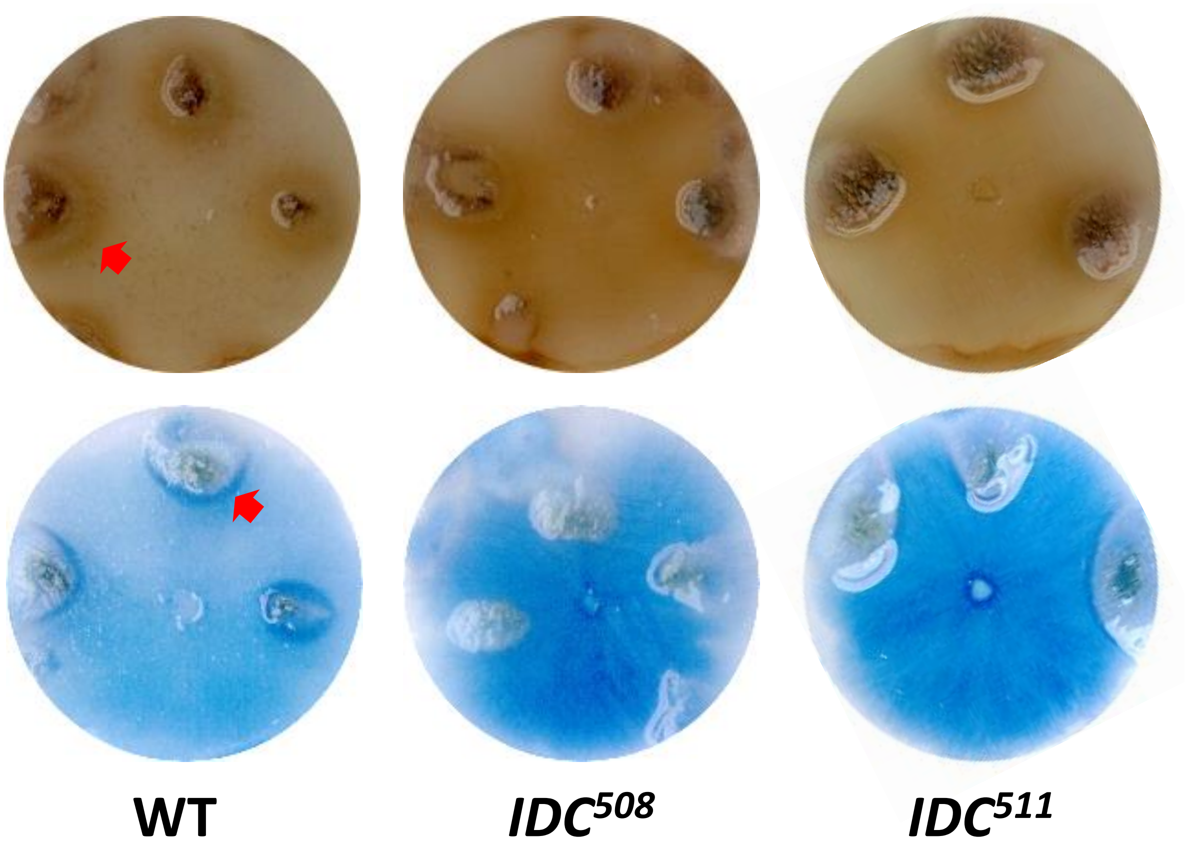

Fig. S4. Hyphal Interference in wild type and mutants. Thallus of *P. anserina* was inoculated at the center and three thalli of *Penicillium chrysogenum* were inoculated 1.5 centimeter away. After three days of incubation, the peroxide burst typical of hyphal interference was visualized by precipitation of diaminobenzidine (Top, arrow shows the halo of peroxide produced by *P. anserina* at the contact with *P. chrysogenum*) and *P. chrysogenum* cell death (bottom, arrow) by coloration with trypan blue .[36]. Both IDC^{508} and IDC^{511} were impaired in the production of peroxide and the killing of *P. chrysogenum*.

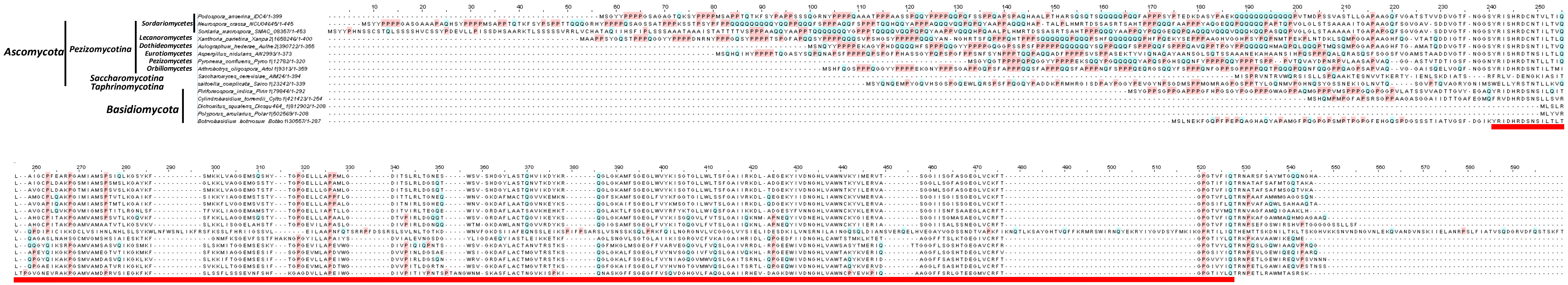

Fig. S5. Alignment of selected IDC4 orthologues/paralogues. The prolines are highlighted in pink and the glutamines in blue. The AIM24 domain is underlined in red.

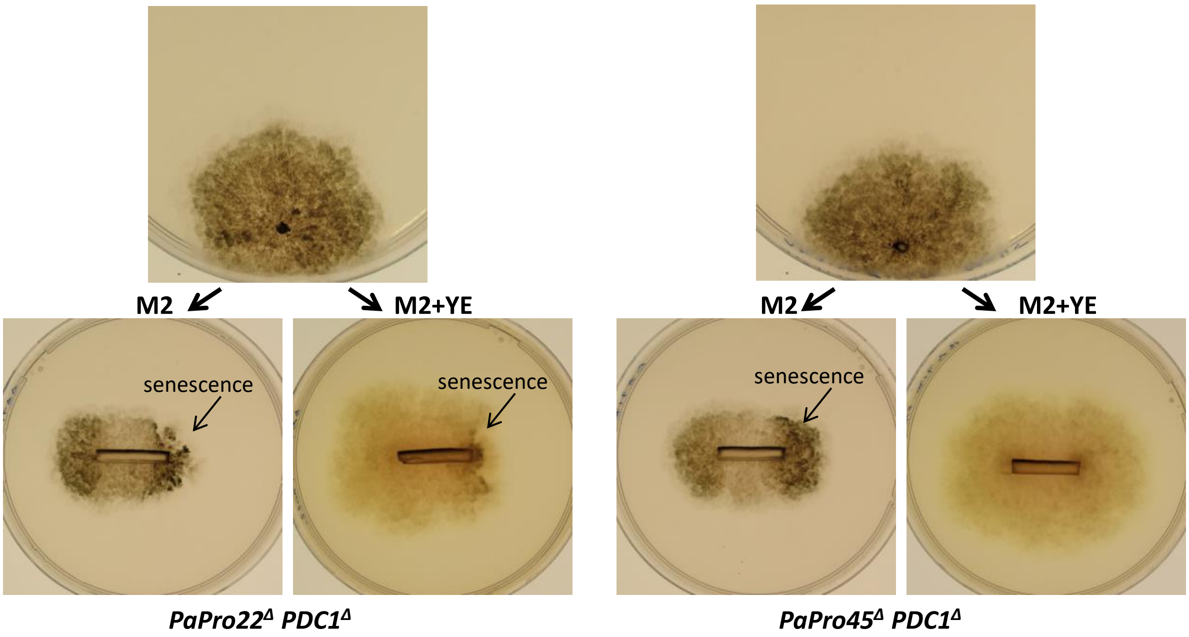

Fig. S6. CG test on in *PaPro22$^{\Delta}$ PDC1$^{\Delta}$* and *PaPro45$^{\Delta}$ PDC1$^{\Delta}$* double mutants. Tests were made as described in Fig. 1 and 8.
