# Supplementary material for: PaPro1 and IDC4, Two Genes Controlling Stationary Phase, Sexual Development and Cell Degeneration in Podospora anserina
Source: J Fungi (Basel). 2018 Jul 11;4(3):85. doi: 10.3390/jof4030085 (PMC6162560; doi:10.3390/jof4030085)
Supplement: Supplementary file 1 [file jof-04-00085-s001.zip › Table S1.pdf]

Table S1

Primers used

**Deletions, tagging and complementation**	
**Primer name**	**sequence**
***IDC4* (Pa_2_230)**	
**Deletion**	
5'Pa_2_230A	aaggcctgtgacacctcacc
5'Pa_2_230B	CTATTTAACGACCCTGCCCTGAACCGttacaagtttccccgcgtt
mk_2_230E	caacgcggggaaacttgtaaCGGTTCAGGGCAGGGTCGTTAAATAG
mk_2_230F	gaccatcaacccgaaccaaaCATCGAACTGGATCTCAACAGCGGTAAG
3'Pa_2_230C	CTTACCGCTGTTGAGATCCAGTTCGATGtttggttcgggttgatggtc
3'Pa_2_230D	ggcttcttgggatggggtag
**Complementation**	
IDC^{508}-left	ggcctgtgacacctcacc
IDC^{508}-right	tctttgtgccaacgacca
**mCherry Tagging**	
XhoI 508 tag for	aaaaactcgagtcacactacaccggtcccg
Hind3 508 tag rev	aaaaaaagcttgggagcatggccgttctgttgtc
***PaPro1* (Pa_1_10140)**	
**Deletion**	
5'Pa_1_10140A	gcacgtcgtacccctgtctc
5'Pa_1_10140B	CTATTTAACGACCCTGCCCTGAACCGcctcgctcgtgttcaaggtc
mk_1_10140E	gaccttgaacacgagcgaggCGGTTCAGGGCAGGGTCGTTAAATAG
mk_1_10140F	gttgtgccccatgatccagtCATCGAACTGGATCTCAACAGCGGTAAG
3'Pa_1_10140C	CTTACCGCTGTTGAGATCCAGTTCGATGactggatcatggggcacaac
3'Pa_1_10140D	cgagaatgggttgactgttgc
***PaPro45* (Pa_1_15490)**	
**Deletion**	
5'Pa_1_15490A	gctaaaagtcgagacagaaaacggtcaagg
5'Pa_1_15490B	CTATTTAACGACCCTGCCCTGAACCGggtaagtcggtggaaaagcgtattcaaaag
mk_1_15490E	cttttgaatacgcttttccaccgacttacccCGGTTCAGGGCAGGGTCGTTAAATAG
mk_1_15490F	atgttgttgtgtgacaaaggaaaacccaagCATCGAACTGGATCTCAACAGCGGTAAG
3'Pa_1_15490C	CTTACCGCTGTTGAGATCCAGTTCGATGcttgggttttcctttgtcacacaacaacat
3'Pa_1_15490D	gaaactctcaaactgcacagcaaccttgac
***PaPro22* (Pa_2_9440)**	
**Deletion**	
5'Pa_1_9440A	atgatttttcagctgcttcaaagttcgtca
5'Pa_1_9440B	CTATTTAACGACCCTGCCCTGAACCGgcatgatcccttgttcacatcaacttcatt
mk_1_9440E	aatgaagttgatgtgaacaagggatcatgcCGGTTCAGGGCAGGGTCGTTAAATAG
mk_1_9440F	cttggatcgtttgctttaccatttaccgacCATCGAACTGGATCTCAACAGCGGTAAG
3'Pa_1_9440C	CTTACCGCTGTTGAGATCCAGTTCGATGgtcggtaaatggtaaagcaaacgatccaag
3'Pa_1_9440D	ggacgaaagtaacgaggattacgtggtgat

**RT-qPCR**					
**Type of gene**[a]	**Gene number**	**Gene name or function**	**Primer name**	**Primer sequence 5'>3'** [b]	**Amplicon size**
HKG	*Pa_1_16650*	*AS1*	AS1f	CAACATGGCTGACGAATAC/AACGC	115 bp
			AS1r	GGAGGTCAGGTCAAGGAGA/GCATC	
HKG	*Pa_3_6780*	*CIT1*	CIT1f	CTCCTCCAAGACCCAG/ACCCTC	100 bp
			CIT1r	GACCTTGGAGCCATGCTCC/TTTC	

HKG	*Pa_3_5110*	*GPD*	GPDf	CATTGAGCCCAAGTACGCT/GAG	113 bp
			GPDr	GTCGCGCTCAGTGTAGAACTTGA	
HKG	*Pa_2_4990*	*HOM2*	HOM2E1E2	GCAAGATGAAGCGCTTCAG/ACTTAC	175 bp
			HOM2E3E2	CGTTTTATTTTGGCGCGT/CTGTTTTG	
HKG	*Pa_2_6460*	*PAH1*	PAH1f	GATCTGGTTCCAGAACCG/ACGTG	247 bp
			PAH1r	GCAGTTGAGATGATGAATCA/AAAC	
HKG	*Pa_7_6690*	*PDF2*	PDFf	GCAGACAGGTTCGAAAAG/ATTG	294 bp
			PDFr	CAGATGATCAATGGTT/TCTTGC	
HKG	*Pa_4_8980*	*TBP*	TBPf	CACACCCACTCTTCA/GAACATT	106 bp
			TBPr	ACGCTTGGGGTTGTA/CTCAGC	
HKG	*Pa_7_8490*	*TIP41*	TIPf	GTTTGCGGAGGTGAAGAAG/AA	146 bp
			TIPr	CCGTCTCACCCTCGAGAC	
HKG	*Pa_4_7790*	*UBC*	UBCf	GGCCATCCCCATCCATCAAC	107 bp
			UBCr	GGTGATGGTCTTGCCAGTGA/GA	
GoI	*Pa_1_13940*	*STE11*	13940AF2	CGGCCCTATTCTAGAT/ATGTC	187 bp
			13940intR	CGGCTTCGCTCTTTCCTTC	
GoI	*Pa_6_4110*	*PaHMG8*	4110f	ACGCTGTCGGATAAGTTTGC	190 bp
			4110r	CCTGTTGCTGCTGCTCCTT	
GoI	*Pa_1_20590*	*FPR1*	FPR1f	GGCGTTCTCAATACAATGAA/GTCG	297 bp
			FPR1r	GCCACCAGTCATGACGGAAATG	
GoI	*Pa_2_2310*	*MFP*	MFPf	CGTACGGGAGTGGACTTGGATGGA	149 bp
			MFPr	CGACACTGAGGCGGTACCCAAAAG	
GoI	*Pa_4_1380*	*PRE2*	1380f	GTTGATGTTTGTGCCCG/TGG	177 bp
			1380r	TCGGTGGTTGTGCCAGTCG	
GoI	N/A[c]	*FMR1*	FMR1f	GGTTTCATGGGCTACCGAT/CCTAC	251 bp
			FMR1r	CATCCAAGGGCTTCCATGTAGC	
GoI	*Pa_1_8290*	*MFM*	MFMf	CCACCCTCGCAACAACACGTTAGA	150 bp
			MFMr	AAACGAAGGCGATGCTCATGTTGG	
GoI	*Pa_7_9070*	*PRE1*	9070f	CGGCGGTCATCTTTACGGT	195 bp
			9070r	GGTAAAAGGTGAGGC/AAGCC	
GoI	*Pa_1_2410*	*PaNox1*	2410f2	GCTGCCCATTACGTCAA/CTTC	94 bp
			2410r2	TGGCACCACCGGGCTGAAC	
GoI	*Pa_1_7250*	*PaNoxD*	7250f1	CCTGGCCTCGGCAACTT/ACC	204 bp
			7250r1	CTGCGGTGGATGCTGGTG	
GoI	*Pa_2_13340*	*PaMpk1*	13340f2	AGGAAAATGCCGGGTACA	112 bp
			13340r2	CCAACAGACCATACGTCAA/TAG	

[a]: HKG: housekeeping gene, used as candidate for reference gene; GoI: gene of interest.

[b]: slash (/) indicates two consecutive exons.
