# Supplementary material for: PaPro1 and IDC4, Two Genes Controlling Stationary Phase, Sexual Development and Cell Degeneration in Podospora anserina
Source: J Fungi (Basel). 2018 Jul 11;4(3):85. doi: 10.3390/jof4030085 (PMC6162560; doi:10.3390/jof4030085)
Supplement: Supplementary file 1 [file jof-04-00085-s001.zip › Table S4.pdf]

mat+

Gene	Type	Reaction Efficiency	Expression	Std. Error	95% C.I.	P(H1)	Result
CIT1	REF	0,88	1,049				
GPD	REF	0,91	0,995				
TBP	REF	0,97	0,959				
STE11	TRG	0,825	1,081	0,892 - 1,314	0,741 - 1,668	0,472	
HMG8	TRG	0,814	0,336	0,256 - 0,422	0,212 - 0,459	0,001	DOWN
FPR1	TRG	0,957	0,403	0,345 - 0,476	0,311 - 0,520	0,000	DOWN
MFP	TRG	0,832	0,169	0,122 - 0,233	0,085 - 0,336	0,000	DOWN
PRE2	TRG	0,969	0,048	0,040 - 0,059	0,034 - 0,066	0,001	DOWN
Nox1	TRG	0,987	0,955	0,898 - 1,009	0,877 - 1,071	0,094	
NoxD	TRG	0,919	0,603	0,530 - 0,680	0,489 - 0,753	0,002	DOWN
Mpk1	TRG	0,983	1,188	1,116 - 1,255	1,043 - 1,279	0,001	UP

mat-

Gene	Type	Reaction Efficiency	Expression	Std. Error	95% C.I.	P(H1)	Result
CIT1	REF	0,88	1,024				
GPD	REF	0,91	0,969				
TBP	REF	0,97	1,007				
STE11	TRG	0,825	1,009	0,784 - 1,294	0,690 - 1,569	0,931	
HMG8	TRG	0,907	0,288	0,220 - 0,383	0,201 - 0,422	0,000	DOWN
FMR1	TRG	0,887	0,145	0,110 - 0,187	0,103 - 0,221	0,000	DOWN
MFM	TRG	0,822	0,306	0,212 - 0,433	0,204 - 0,509	0,000	DOWN
PRE1	TRG	0,972	0,033	0,025 - 0,046	0,019 - 0,055	0,001	DOWN
Nox1	TRG	0,987	1,092	1,013 - 1,186	0,996 - 1,262	0,006	UP
NoxD	TRG	0,919	0,624	0,566 - 0,679	0,543 - 0,716	0,000	DOWN
Mpk1	TRG	0,983	1,274	1,136 - 1,450	1,073 - 1,592	0,001	UP

Cq and efficiencies used for these analyses are in Table_SI_2_Cq.
